# Supplementary material for: Recovery of Lutacidiplasmatales archaeal order genomes suggests convergent evolution in Thermoplasmatota
Source: Nat Commun. 2022 Jul 15;13:4110. doi: 10.1038/s41467-022-31847-7 (PMC9287336; doi:10.1038/s41467-022-31847-7)
Supplement: Supplementary file 1 — Supplementary Information [file 41467_2022_31847_MOESM1_ESM.pdf]

Supplementary Information: Recovery of Lutacidiplasmatales archaeal order genomes suggests convergent evolution in Thermoplasmatota

Paul O. Sheridan[1,2], Yiyu Meng[1], Tom A. Williams[2], Cécile Gubry-Rangin[1,#]

Supplementary Methods

Extended Phylogenomics Short name: SI Phylogenomics Methods

Detailed description of genome datasets

This study used three datasets to build a full Thermoplasmatota tree, a Thermoplasmatota tree containing only higher-quality genomes, and a tree restricted to the Lutacidiplasmatales.

The **full dataset** tree comprises 124 archaeal genomes with completeness greater than 45% and less than 10% contamination. This dataset contains the 35 newly sequenced genomes, the TMEG-bg1 genome, 84 genomes representing 84 other Thermoplasmatota species and 4 Archaeoglobales genomes (the outgroup).

The **higher-quality dataset** tree comprises 100 archaeal genomes with completeness greater than 70% and less than 5% contamination. This dataset contains 21 newly sequenced genomes, the TMEG-bg1 genome, 74 genomes representing 74 other Thermoplasmatota species and four Archaeoglobales genomes.

The **Lutacidiplasmatales-specific dataset** comprises 40 archaeal genomes with completeness greater than 45% and less than 10% contamination. This dataset contains 35 newly sequenced genomes, two publicly available Lutacidiplasmatales genomes and 3 Methanomassiliicoccales genomes (the outgroup).

Sulfite oxidation genes

Protein sequences possessing the Pfam oxidoreductase molybdopterin binding domain (PF00174) were downloaded from Swiss-Prot. They were combined with sequences containing the PF00174 conserved domain from the full Thermoplasmatota genome dataset (this study) and from the Thaumarchaeota genome dataset (Sheridan et al. 2020[1]), resulting in 213 sequences. Conserved domains within the sequences were annotated using InterProScan [2], Pfam [3], SUPERFAMILY [4] and MobiDBLite [5] databases. Transmembrane helices were predicted using TMHMM 2.0 [6].

The 213 sequences were then aligned using MAFFT L-INS-i [7], processed with trimAl (automated1)[8], and an ML phylogenetic tree was constructed using IQ-TREE 2.0.3[9] with 2,000 UFBoot replicates and 1,000 SH-aLRT test[10], an NNI search and the best substitution model selected by ModelFinder[11]). The ancestral deviation for each node was calculated using MAD programme[12], and the node with the minimal ancestor deviation was used as the tree's root.

Supplementary Results

A robust phylogeny for the Thermoplasmatota. Short name: SI Phylogenomics Results

The increased availability of genomes in recent years and the new genomes sequenced in this study allow revisiting deep evolutionary relationships within the Thermoplasmatota. Eight phylogenomic trees were created to estimate the phylogeny of the Thermoplasmatota using different approaches. Seven of the eight species trees constructed in this work for Thermoplasmatota differed to some extent from the topology presented in Adam *et al.* 2017[13] (a thorough investigation spanning multiple archaeal phyla) (Tree 7, Topology B, Supplementary Fig. 4) and some other works[14, 15]. Six trees (Trees 1-6, Topology A, Supplementary Fig. 4) resolved Acidiprofundales and Thermoplasmatales as a basal monophyletic group in the Thermoplasmatota. In contrast, the remaining tree (Tree 8, Topology C, Supplementary Fig. 4) implied an internal branching of this group (albeit with very poor support). Statistical analysis of the three differing topologies strongly favoured Topology A over Topologies B and C. Therefore, trees with this Topology A were used in further evolutionary analysis – Tree 1 was used in Figure 2 and Tree 4 was used for all gene tree-species tree reconciliation work. Marker gene information for the full dataset trees is provided in Supplementary Data 20.

Distribution of posterior probabilities in predictions of gene family origination events

The likelihood of each gene family originating a single time into the Thermoplasmatota was estimated for every candidate originating branch (Supplementary Data 21). With a 0.5 posterior probability (PP) criterion on a single branch, over 70% (4,256 of 6,050) of the gene families were predicted to have been acquired a single time into the Thermoplasmatales. This percentage declined to 60, 50, 38, 25 and 9 % when the threshold was increased to PPs greater than 0.6, 0.7, 0.8, 0.9 and equal to 1.0, respectively. Therefore, even at the permissive criterion (0.5 PP), a single point of origination could not be predicted for almost 30 % of the gene families used in the gene tree – species

tree reconciliation analysis, and this number increased notably as the PP threshold was made more stringent (Supplementary Fig. 25).

Origination and evolution of complex IV assembly components ctaA, ctaB and coxB. Short name: SI Complex IV evolution.

The *cta*A genes detected in Lutacidiplasmatales and Poseidonales were highly divergent from each other (Supplementary Fig. 11), indicating two independent acquisitions of *cta*A into the Thermoplasmatota. The *cta*A genes of Poseidonales were potentially acquired from cyanobacteria, given their *cta*A close phylogenetic relationship and their shared marine environment. In contrast, the Lutacidiplasmatales *cta*A genes are affiliated with various bacterial lineages with no discernible shared environment.

The *cta*B gene, which is responsible for the biosynthesis of haem O from haem B, appears to have a more complicated evolutionary history. Again, the Poseidonales genes diverged from the other Thermoplasmatota and cluster more closely with bacterial homologs (Supplementary Fig. 12), indicating independent originations into Thermoplasmatota. In addition, the three orders, Thermoplasmatales, Lunaplasmatales and Lutacidiplasmatales, were all separated into two clades. Gene tree - species tree reconciliation indicates that this ancestral splitting did not occur through an ancient duplication event. Therefore, it is likely that there were at least three independent origins of *cta*B in the Thermoplasmatota: one ancestral gene present in Thermoplasmatales, one gene which has possibly been acquired from the Thaumarchaeota and is present in Thermoplasmatales, Lutacidiplasmatales and Lunaplasmatales, and a third of bacterial origin into the Poseidonales (Supplementary Fig. 12). The presence of *cta*B in SAL16 and TMEG-bg1 is suggested by gene tree - species tree reconciliation to have most likely (albeit with weak support) occurred by transfer from the Thermoplasmatales LCA to SAL16 (0.31 TPP (posterior probability of transfer)) and then subsequently from SAL16 to the LCA of TMEG-bg1 and UBA184 (0.26 TPP) (Supplementary Data 22).

In contrast to the other subunits of the complex IV, The *cox*B genes of Lutacidiplasmatales, Poseidoniales, Lunaplasmatales and most Thermoplasmatales genomes cluster together. At the same time, a Thermoplasmatales clade, consisting of the genera *Ferroplasma*, *Acidiplasma* and *Picrophilus,* possess divergent *cox*B genes more similar to that of *Halobiforma lacisalsi* (Supplementary Fig. 12). The reason for this is unknown, but it is noteworthy that several of the organisms that possess this divergent *cox*B are acidophilic ferrous iron oxidisers from distantly related microbial lineages.

Progressive evolutionary history of Lutacidiplasmatales

The evolution of Lutacidiplasmatales from the Thermoplasmatota LCA is predicted to have been marked by at least three bifurcating divergences. Functional gene gain and loss were analysed by comparing progressive ancestral genome reconstructions (Supplementary Fig. 26) and validated by origination posterior probability (OPP) if the gene family is predicted to have originated only once in Thermoplasmatota.

The first bifurcating divergence in this analysis formed a Thermoplasmatota clade, TP_2 LCA, excluding o_Acidiprofundales and o_Thermoplasmatales. This divergence coincided with the gain of gene families, including a nickel-containing superoxide dismutase, a K+ stimulated pyrophosphate-energised sodium pump and several amino acid metabolism genes, and the loss of gene families including the Pgi1-type glucose-6-phosphate isomerase.

The second divergence exclude the o_Poseidonales and o_Thermoprofundales. This divergence coincided with the gain of genes such as a divergent form of the oxidative protective peptide methionine sulfoxide reductase and phosphoglycolate phosphatase, which prevents the inhibition of glycolysis by phosphoglycolate. It also coincided with the loss of genes such as poorly characterised Archaeal PilT-family ATPase.

The third divergence comprises only the Lutacidiplasmatales LCA, excluding the rest of the Thermoplasmatota lineages. This divergence coincided with one of the most significant gene family gains by origination detected in the Thermoplasmatota. Several genes involved in glycolysis were gained, including ATP-dependent phosphofructokinase *pfk*, and fructose-bisphosphate aldolase class I, *fba*B (0.97 OPP). Several gene families involved in oxidative phosphorylation were also acquired, including the originating gene families heme A synthase, *cta*A, and heme-copper oxygen reductase subunit b, *cox*B. Additionally, this divergence coincided with the origination of heterotrophy genes such as the sarcosine oxidase subunits A (0.87 OPP) and B (0.99 OPP) and the gain of pentose phosphate pathway genes glucose-6-phosphate 1-dehydrogenase, *zwf*, and 6-phosphogluconate dehydrogenase, gnd. The third divergence also included some notable losses, such as the three genes involved in the biosynthesis of histidine (*his*B, F and G) from products of the pentose phosphate pathway and the loss of indolepyruvate ferredoxin oxidoreductase (both A and B subunits lost), an enzyme involved in archaeal peptide fermentation[16]. The expanded functional gene gain and loss in the evolution from the Thermoplasmatota LCA to Lutacidiplasmatales LCA can be found in Supplementary Data S23.

Gene families predicted to have originated in the LCA of Lutacidiplasmatales were queried against the UniRef90 database using the permissive criteria (E-value $1x10^{-3}$). Homologs were detected for 81 % of the originating gene families. However, over half of these hits were classified with terms such as "Thermoplasmata archaeon" and "Euryarchaeota_archaeon" or "Mine drainage metagenome", which is an environment where Lutacidiplasmatales is known to be prevalent, from the 16S rRNA gene analysis shown earlier. There was, therefore, a significant chance of some of the hits being for genes from Lutacidiplasmatales or other Thermoplasmatota species. Therefore, UniRef90 was filtered for genes with strain-level classifications and genes from Thermoplasmatota species were removed. Homologs were detected for 55 % of gene families originating in the LCA of Lutacidiplasmatales using the filtered database (Supplementary Data 24). Fifty-one per cent of these hits were for genes from bacteria, and 49 % were for genes from archaea. Notably, 10% of the hits were for genes from a single organism, the soil archaeon Thorarchaeota strain OWC[17] (Supplementary Data 24).

Rooting the sulfite oxidase family with minimal ancestor deviation (Short name: MAD rooting)

The ancestor deviation was predicted for every branch of the sulfite oxidase phylogenetic tree using the minimal ancestor deviation approach[12]. The five branches containing the slightest ancestor deviation were used to root the tree, and the topology was inspected for each. Each of the rooted trees supports a clade comprising the eukaryotic sulfite oxidase and nitrate reductase, and the Thaumarchaeota and Lutacidiplasmatales putative sulfite oxidases to the exclusion of the other members of the family (Supplementary Fig. 2), indicating a common ancestry spanning domains of life.

Duplication and loss of gene families originating in Lutacidiplasmatales in comparison to ancestral gene families (Short name: Gene duplication)

Increased duplication and loss rates were observed in laterally acquired gene families (in comparison to ancestral gene families) in Nitrososphaerales lineages[1]. In contrast, the gene families acquired by the Lutacidiplasmatales LCA generally have lower rates of duplication and loss in Lutacidiplasmatales lineages when compared to ancestral gene families (Supplementary Data 25). In Nitrososphaerales, these lineage-specific duplications and losses were theorised to have enabled these organisms to occupy refined niches within terrestrial and sediment environments. The lack of these changes in Lutacidiplasmatales may indicate that the extant members of the order occupy a similar niche to their LCA. However, it is worth noting that all of the Lutacidiplasmatales genomes in this study are from soil and that sequences with similarity to this group have been found in a myriad of

non-soil environments. Therefore, increased taxon sampling from different environments may reveal this order's gene content evolutionary history to be more complex than predicted here.

Supplementary Figures

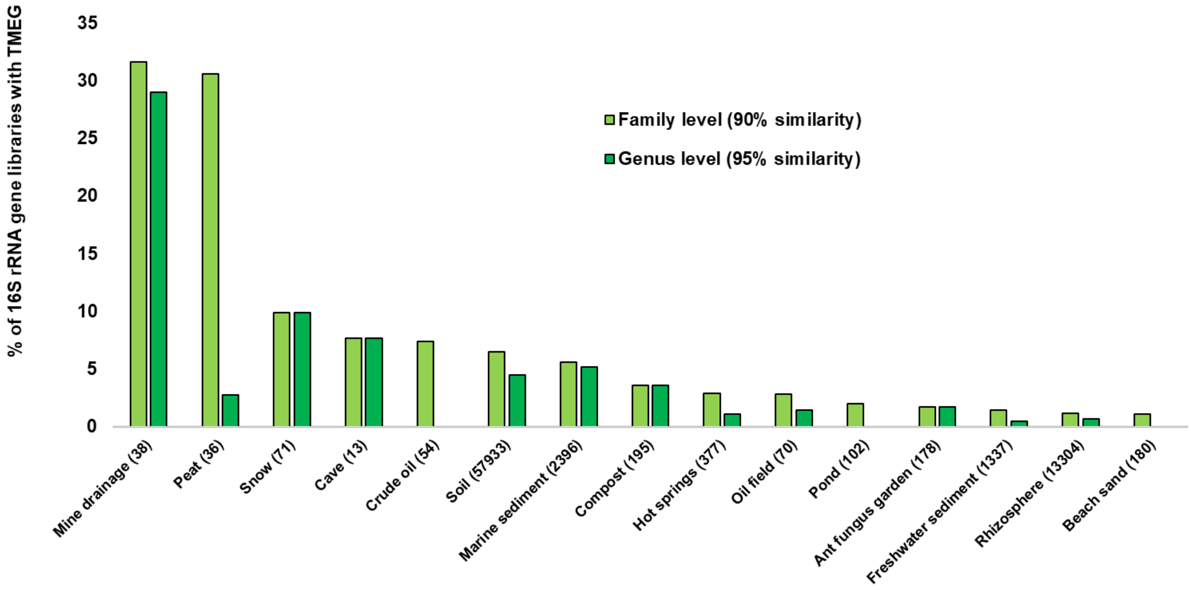

Supplementary Fig. 1. Distribution of TMEG in publicly available 16S rRNA gene libraries from many environments. The 16S rRNA gene of AcS3-62 was queried against the extensive collection of 16S rRNA libraries in IMNGS[18] for reads of ≥ 400 bp that possessed ≥ 90 % sequence similarity.

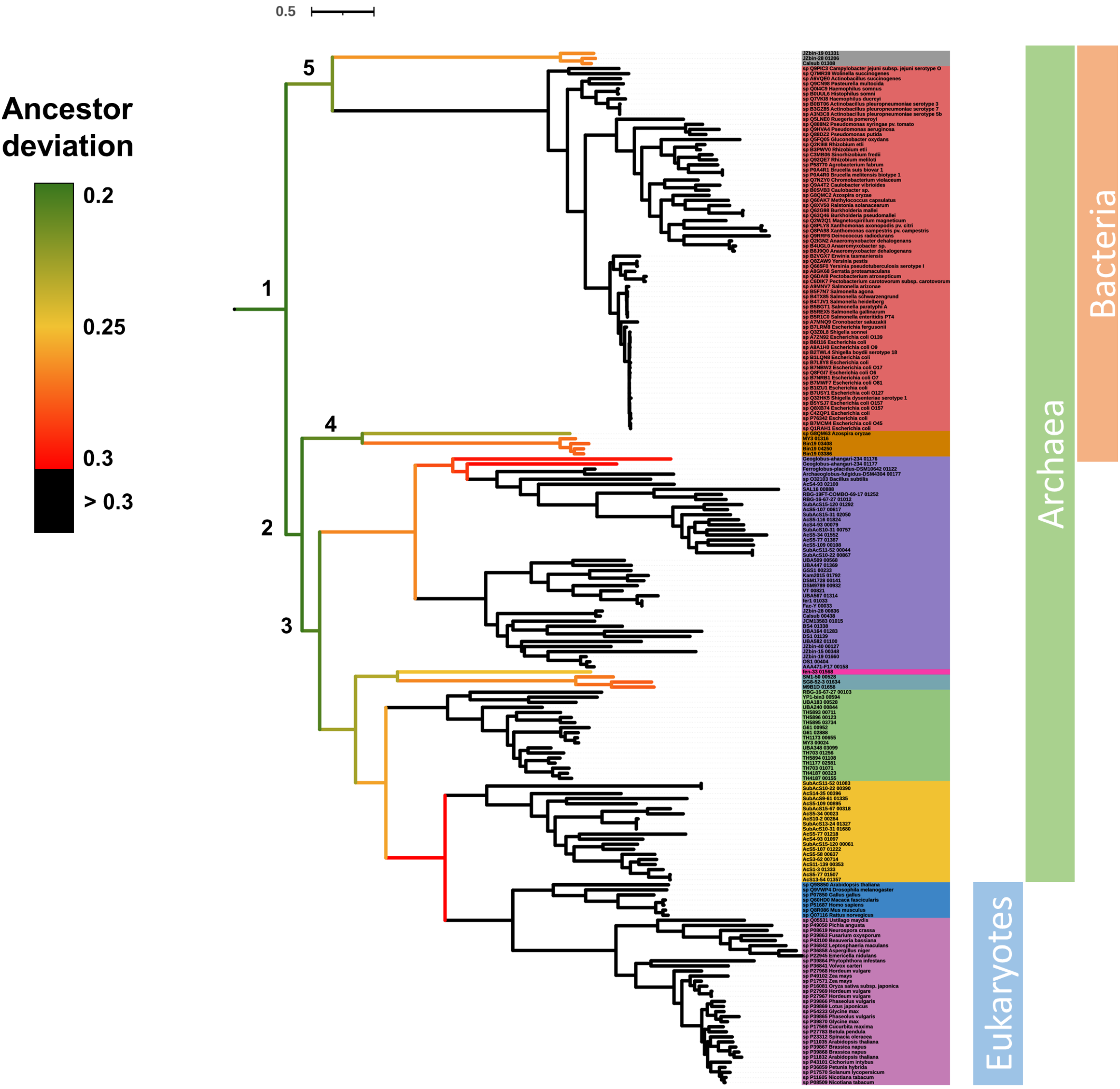

Supplementary Fig. 2. Ancestor deviation in candidate roots of the sulfite oxidase family tree. Numbers indicate a rank of the most favoured roots by minimal ancestor deviation approach (**1** being the most favoured root). These roots show that the Lutacidiplasmatales sulfite oxidases are the prokaryotic sequences most closely related to eukaryotic sulfite oxidases and nitrate reductases.

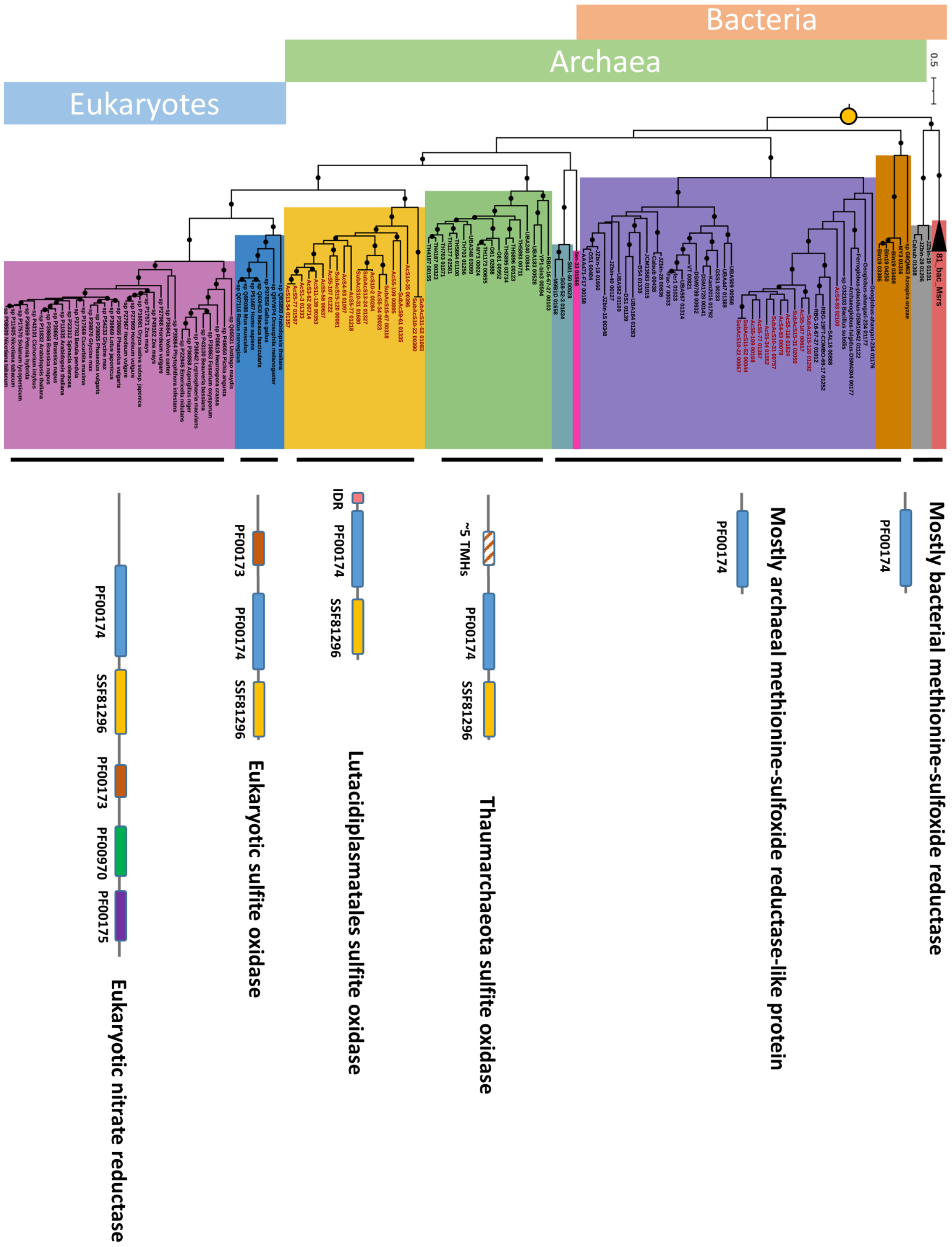

Bacteria
Archaea
Eukaryotes
0.5
81_bac_Msrs
Mostly bacterial methionine-sulfoxide reductase
PF00174
Mostly archaeal methionine-sulfoxide reductase-like protein
PF00174
Thaumarchaeota sulfite oxidase
~5 TMHs
PF00174
SSF81296
Lutacidiplasmatales sulfite oxidase
IDR
PF00174
SSF81296
Eukaryotic sulfite oxidase
PF00173
PF00174
SSF81296
Eukaryotic nitrate reductase
PF00174
SSF81296
PF00173
PF00970
PF00175

Supplementary Fig. 3. Evolution of a putative archaeal class of sulfite oxidases. Phylogenetic tree containing the sulfite oxidase superfamily members and a schematic of their conserved domains. Dots indicate branches with ≥ 70% of 2,000 UFBoot and 1,000 SH-aLRT replicates. Swiss-Prot sequences possessing the PF00174 domain and the previously identified Thaumarchaeota members of this family[1] were included in the analysis. The 81_bac_Msrs clade contains 81 bacterial methionine-sulfoxide reductase genes. Coloured bars on the left indicate the broad taxonomic affiliation of the protein clades. Overlapping of these bars indicates clades with proteins from more than one domain of life. Figures to the right of the tree are schematic representations of domain organisation in the corresponding protein clade with the following nomenclature: Transmembrane helices (TMHs) and Intrinsic disorder region (IDR), i.e. a natively unfolded region. The tree was rooted with minimal ancestor deviation (MAD), as described in Supplementary Fig. 2.

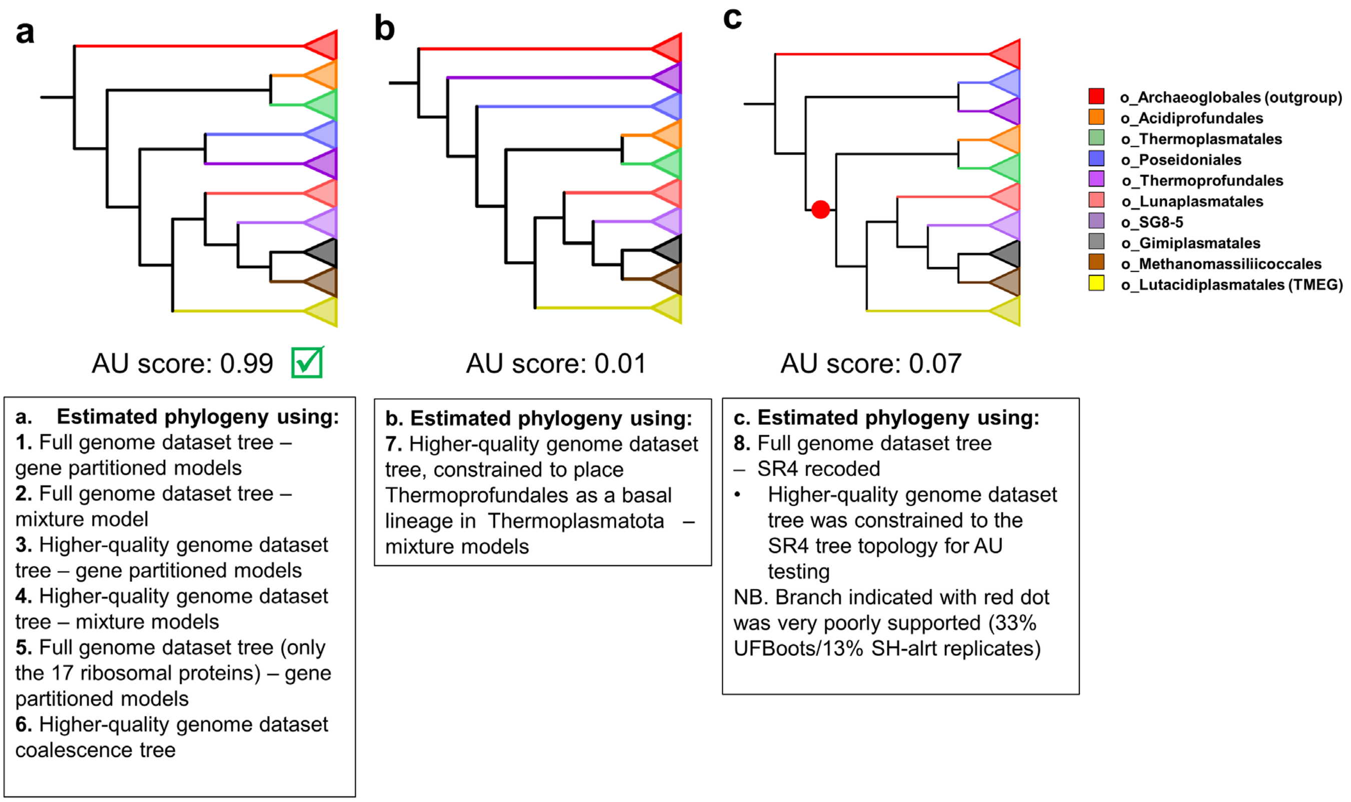

Supplementary Fig. 4. Thermoplasmatota species tree estimation using multiple approaches and comparison of differing resulting topologies. Eight trees were reconstructed, using eight approaches: the six approaches listed in box "a" resulted in the topology presented in panel "a"; the approach listed in box "b" resulted in the topology presented in panel "b"; the approach listed in box "c" resulted in the topology presented in panel "c". Approximately unbiased (AU) testing of the unconstrained Higher-quality genome dataset – gene partitioned models tree "3" with the topology "a" and constrained trees "7" and "8" with the topologies of "b" and "c", respectively. Topology "a" received the highest likelihood and is the favoured hypothesis, although topology "c" could not be rejected at the $P < 0.05$ level. The red dot represents a poorly supported branch indicating an internal placement of Acidiprofundales and Thermoplasmatales in the Thermoplasmatota.

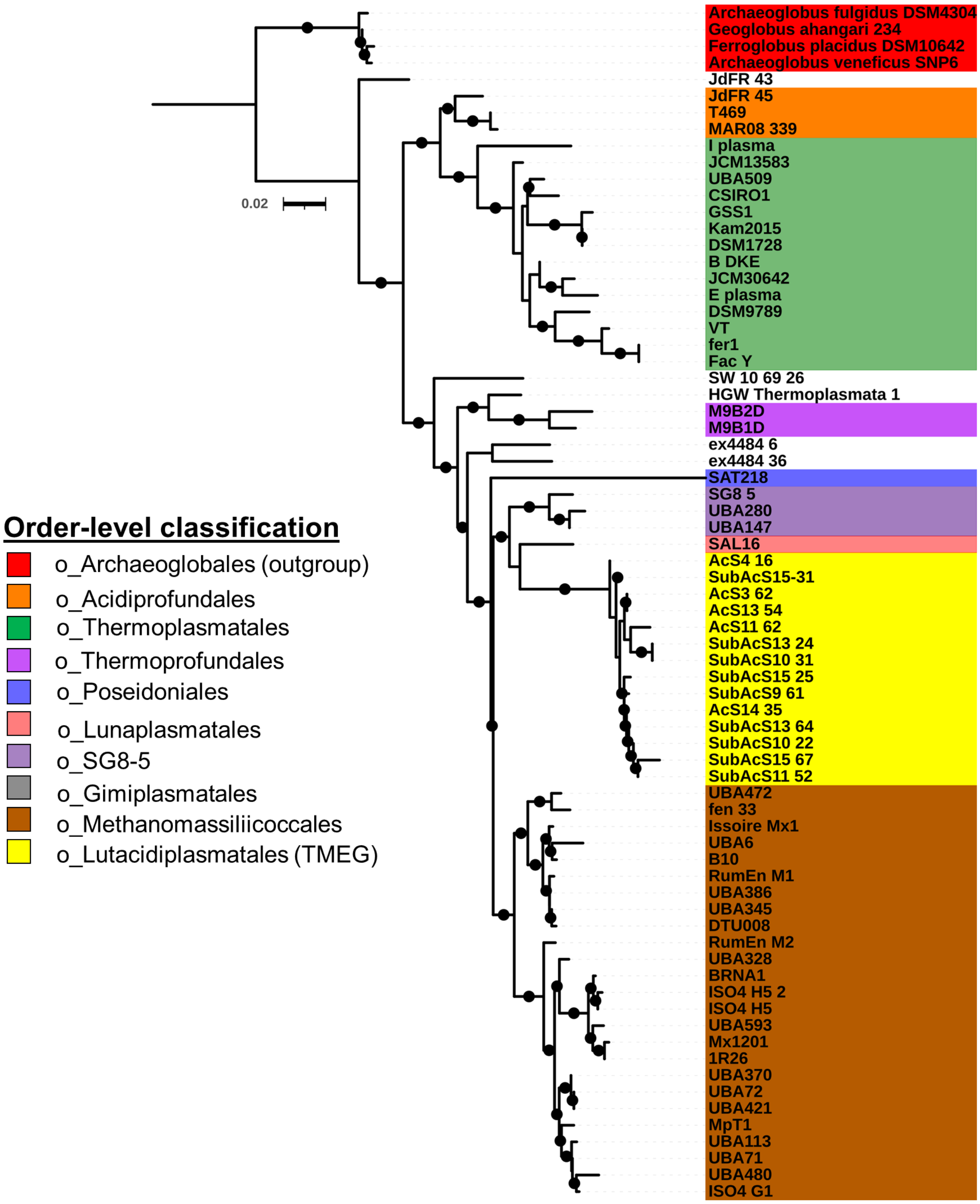

Supplementary Fig. 5. Phylogenetic 16S rRNA gene tree of Thermoplasmatota genomes. 16S rRNA gene sequences of ≥ 450 bp were extracted from the genomes analysed in this study. The final nucleotide alignment possessed 956 columns, 267 of which were parsimoniously informative. Dots indicate branches with ≥ 70% of 1,000 UFBoot replicates. The tree is rooted with the Archaeoglobales genes.

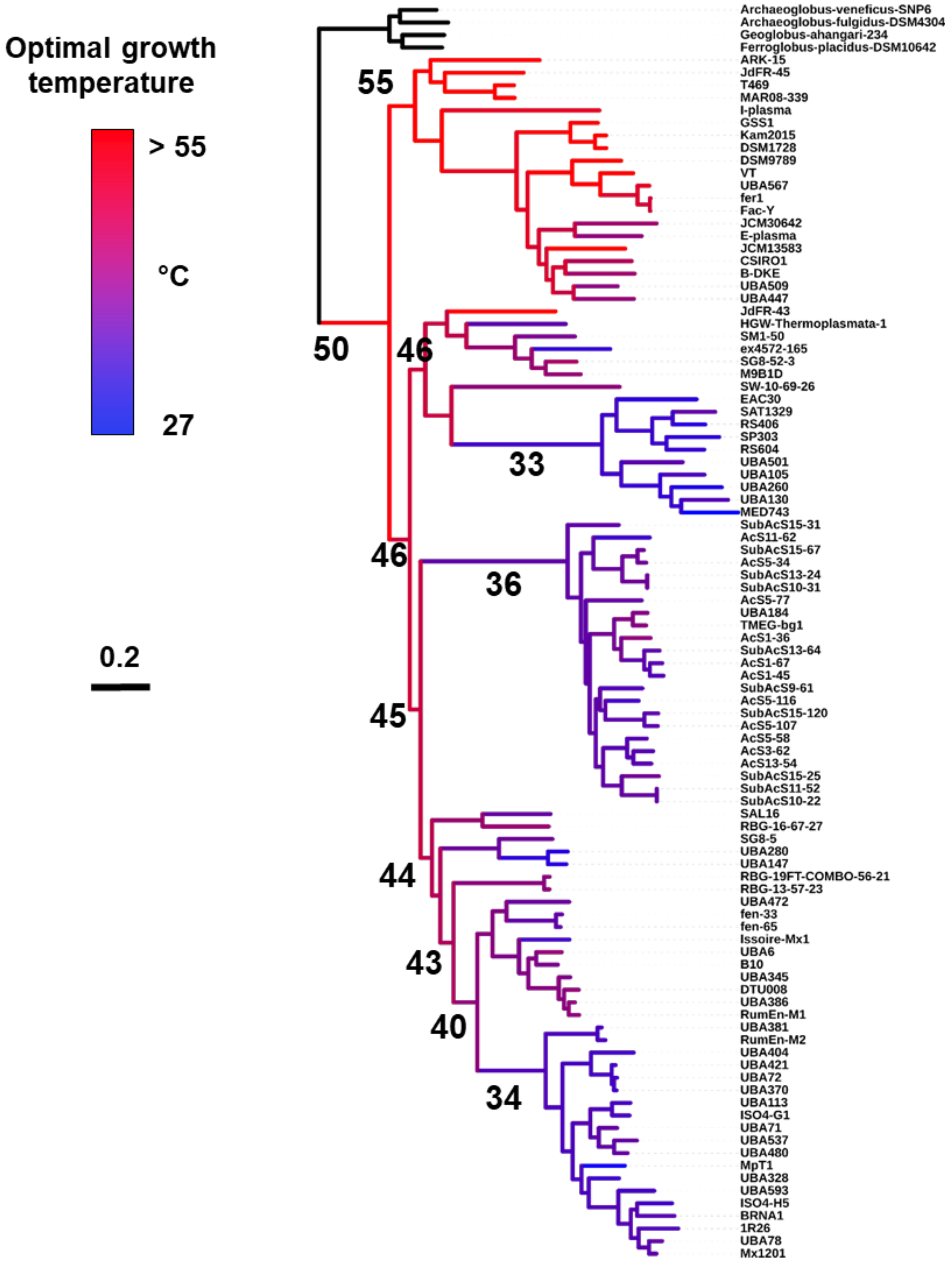

Supplementary Fig. 6. Sequence-based prediction of thermal adaptation throughout Thermoplasmatota history. Tome[19] was used to estimate the optimal growth temperatures (OGT) of extant Thermoplasmatota genomes. Ancestral OGTs were predicted based on a ridge regression approach[20]. Branches were coloured based on their predicted OGT, and key ancestral OGTs were specified on specific branches.

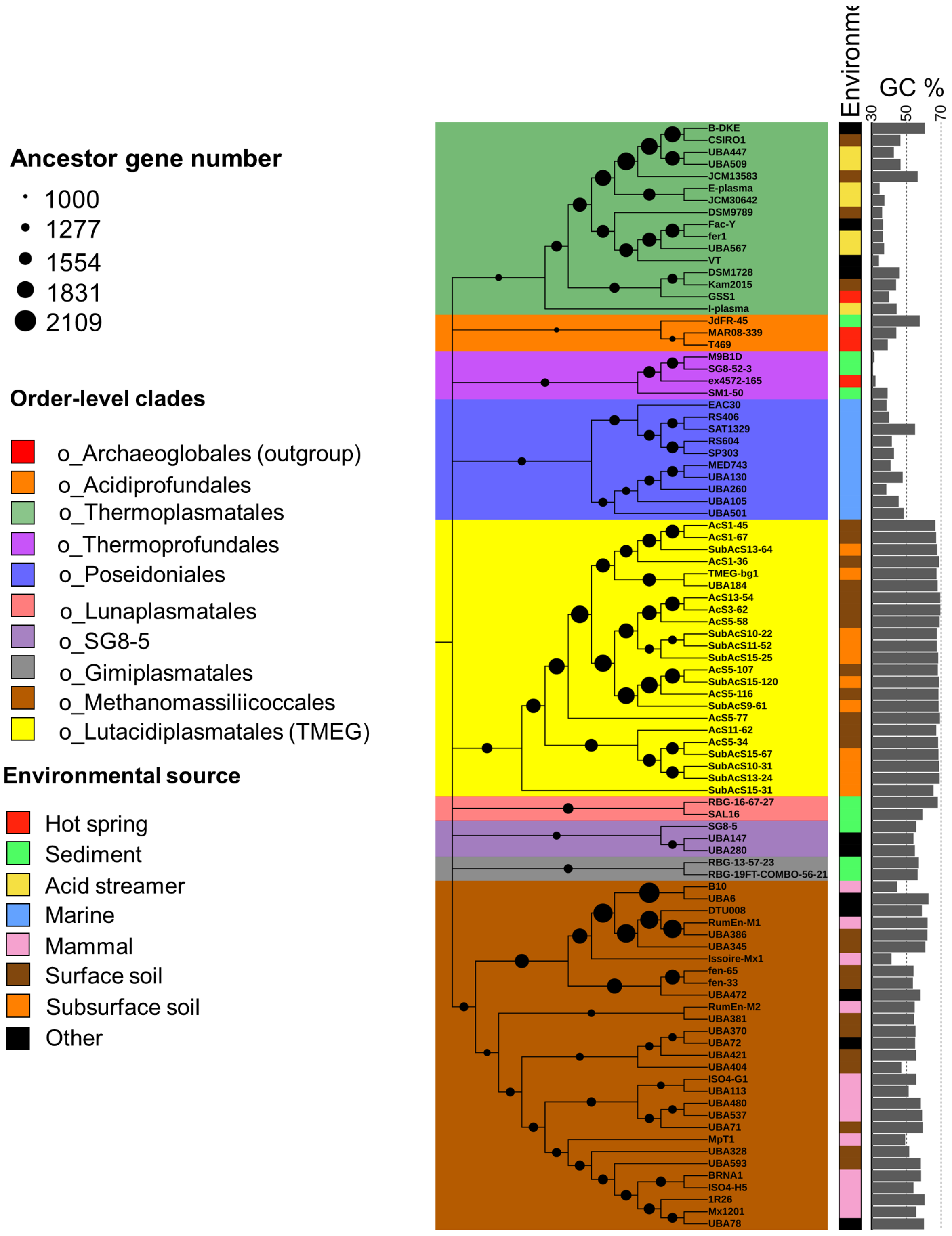

Supplementary Fig. 7. Proteome size evolution and GC-content in Thermoplasmatota. Colours across the phylogenetic tree indicate the order-level taxonomic affiliation of genomes. Dots sizes on branches represent the number of genes predicted in each ancestor reconstruction by gene tree – species tree reconciliation.

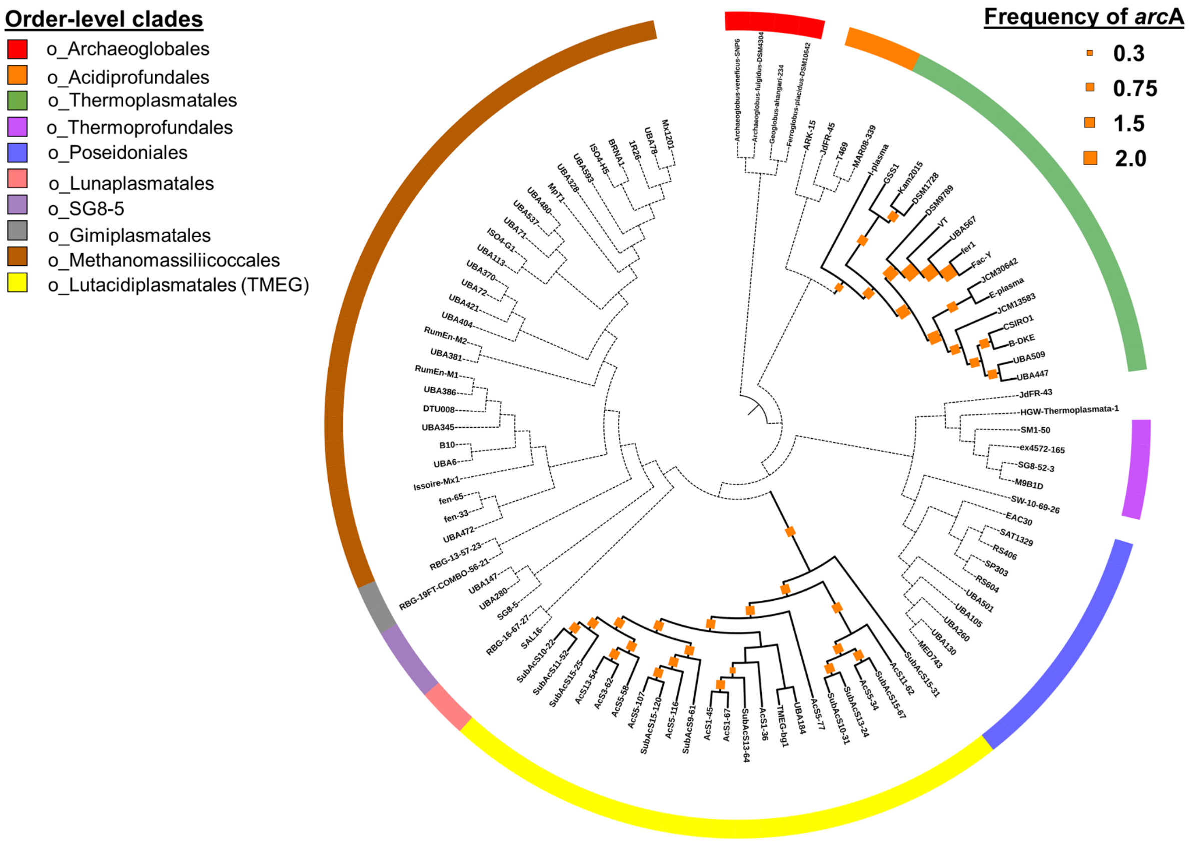

Supplementary Fig. 8. Ancestral gene content comparison to detect multiple lateral acquisitions of the same gene family. Gene tree-species tree reconciliation infers the frequency of a gene family on each branch of the species tree. Multiple lateral acquisitions (from inside or outside the phylum) were predicted when various lineages, but not the last common ancestor of these clades, possess a given gene family. In the example above, the arginine deiminase *arc*A gene family is present in the Lutacidiplasmatales and Thermoplasmatales lineages but is not present in Thermoplasmatales last common ancestor. This indicates *arc*A entered these orders by separate lateral gene transfers rather than vertical inheritance from a common ancestor. Individual gene trees with a diverse representation of *arc*A genes from multiple phyla can then be used to infer whether both lateral gene transfers were from different phyla or whether *arc*A was transferred between the Lutacidiplasmatales and Thermoplasmatales.

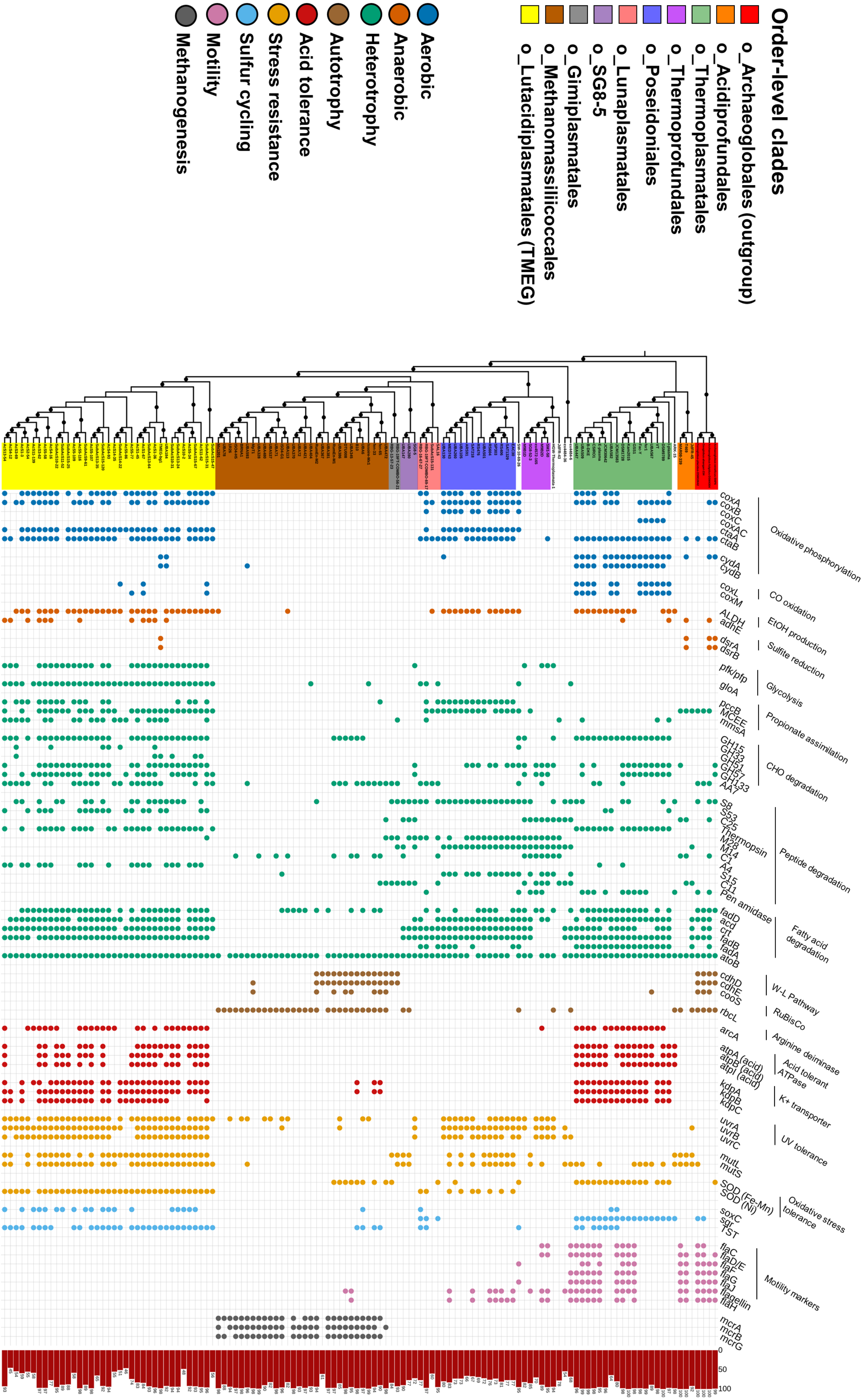
Order-level clades
o_Archaeoglobales (outgroup)
o_Acidiprofundales
o_Thermoplasmatales
o_Thermoprofundales
o_Poseidoniales
o_Lunaplasmatales
o_SG8-5
o_Gimiplasmatales
o_Methanomassiliicoccales
o_Lutacidiplasmatales (TMEG)
Aerobic
Anaerobic
Heterotrophy
Autotrophy
Acid tolerance
Stress resistance
Sulfur cycling
Motility
Methanogenesis
Oxidative phosphorylation
CO oxidation
EtOH production
Sulfite reduction
Glycolysis
Propionate assimilation
CHO degradation
Peptide degradation
Fatty acid degradation
W-L Pathway
RuBisCo
Arginine deiminase
Acid tolerant ATPase
K+ transporter
UV tolerance
Oxidative stress tolerance
Motility markers

Supplementary Fig. 9. Metabolism of the Thermoplasmatota. The presence of selected genes was assessed for all genomes in the analysis. *cox*A (heme-copper oxygen reductase, subunit A; K02274), *cox*B (heme-copper oxygen reductase, subunit B; K02275), *cox*C (heme-copper oxygen reductase, subunit C; PF00510), *cox*AC (heme-copper oxygen reductase, subunit AC; K15408), *cta*A (heme a synthase; K02259), *cta*B (heme o synthase; K02257), *cyd*A (cytochrome bd ubiquinol oxidase, subunit A; K00425), *cyd*B (cytochrome bd ubiquinol oxidase, subunit B; K00426), *cox*L (aerobic carbon-monoxide dehydrogenase, large subunit; K03520), *cox*M (aerobic carbon-monoxide dehydrogenase, medium subunit; K03519), ALDH (aldehyde dehydrogenase (NAD+); K00128), *adh*E (alcohol dehydrogenase; K0407), *dsr*AB (dissimilatory sulfite reductase, subunits A and B; K11180 and K11181), *pfk/pfp* (ATP-dependent phosphofructokinase; K21071), *glo*A (lactoylglutathione lyase; K01759), *pcc*B (propionyl-CoA carboxylase, beta chain, K01966), MCEE (methylmalonyl-CoA/ethylmalonyl-CoA epimerase; K05606), *mms*A (methylmalonate-semialdehyde dehydrogenase; K00140), GH# (glycoside hydrolase family #; dbCAN), Pen amidase (Penicillin amidase), *fad*D (long-chain acyl-CoA synthetase; K01897), *acd* (acyl-CoA dehydrogenase; K00249), *crt* (enoyl-CoA hydratase; K01715), *fad*B (3-hydroxybutyryl-CoA dehydrogenase; K00074), *fad*A (acetyl-CoA acyltransferase; K00632), *ato*B (acetyl-CoA C-acetyltransferase; K00626), *cdh*DE (acetyl-CoA decarbonylase/synthase complex D; K00194 and E; K00197 subunits), *coo*S (anaerobic carbon-monoxide dehydrogenase catalytic subunit; K00198), *rbc*L (ribulose-bisphosphate carboxylase large chain; K01601), *arc*A (arginine deiminase; K01478), *atp*ABI (acid) (V/A-type atpase A, B and I subunits; K02117, K02118 and K02123, plus gene tree analysis), *kdp*ABC (K+ transporting ATPase subunits A, B and C; K01546, K01547 and K01548), *uvr*ABC (excinuclease subunits A, B and C; K03701, K03702 and K03703), *mut*LS (DNA mismatch repair proteins L and S; K03572 and K03555), SOD (Fe-Mn) (superoxide dismutase Fe-Mn family; K04564), SOD (Ni) (nickel superoxide dismutase; K00518), *sox*C (sulfite oxidase; TIGR04555), *sqr* (sulfide:quinone oxidoreductase; K17218), TST (thiosulfate/3-mercaptopyruvate sulfurtransferase; K01011), *fla#* (archaellum subunits C; arCOG05119, D/E ; arCOG02964, F; arCOG01824, G; arCOG01822 and J; arCOG01809), flagellin (archaeal flagellin; PF01917) and *fla*H (archaellum subunits C;PF06745) and *mrc*ABG (methyl-coenzyme M reductase A; K00399, B; K00401, C; K00402 subunits). The predicted completeness of each genome sequence is indicated in the far right red bar chart.

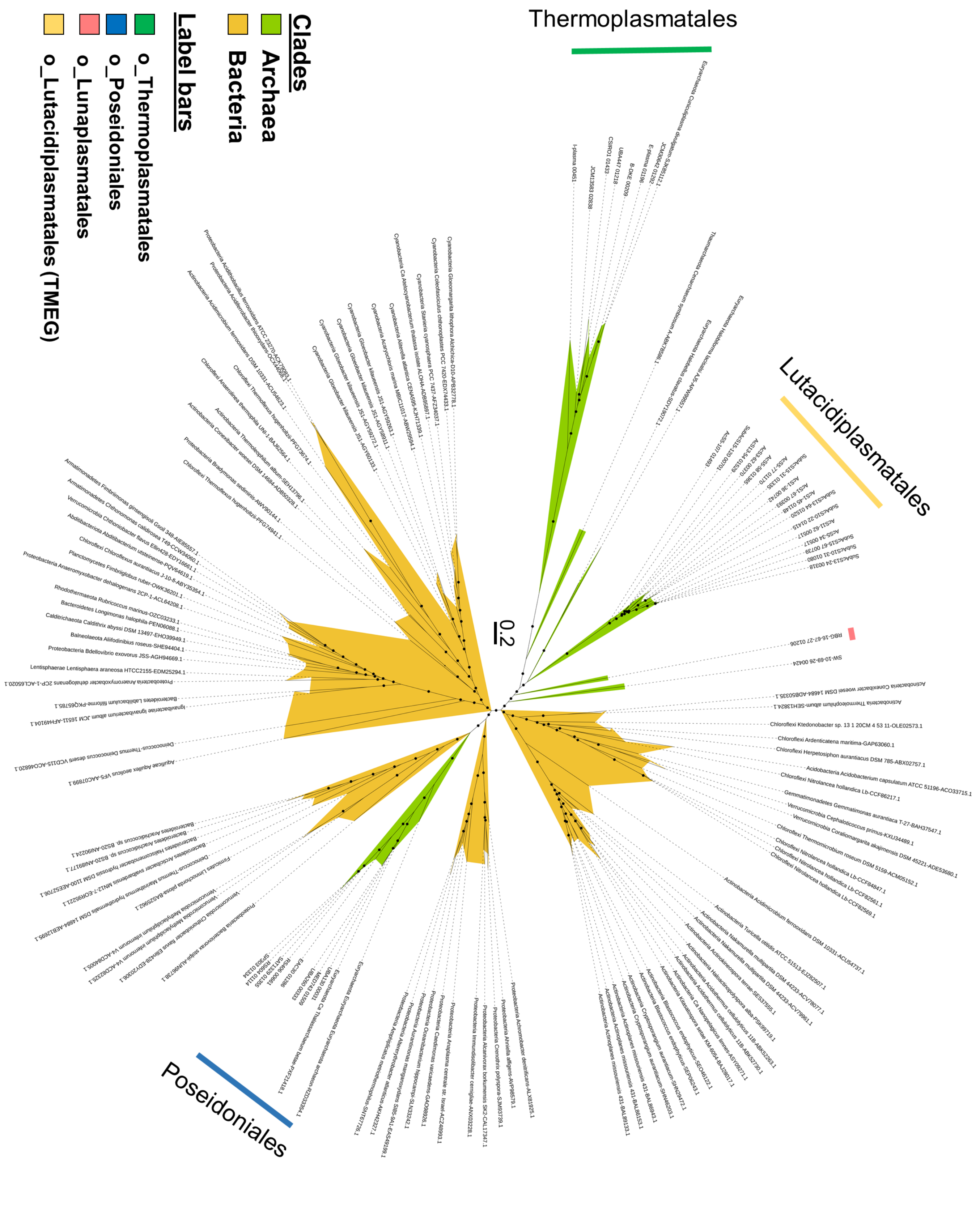

Supplementary Fig. 10. Phylogeny of the heme-copper oxygen reductase subunit A (*cox*A) gene. Dots indicate branches with ≥ 70% of 1,000 UFBoot replicates. The tree was estimated using the model LG+F+R7.

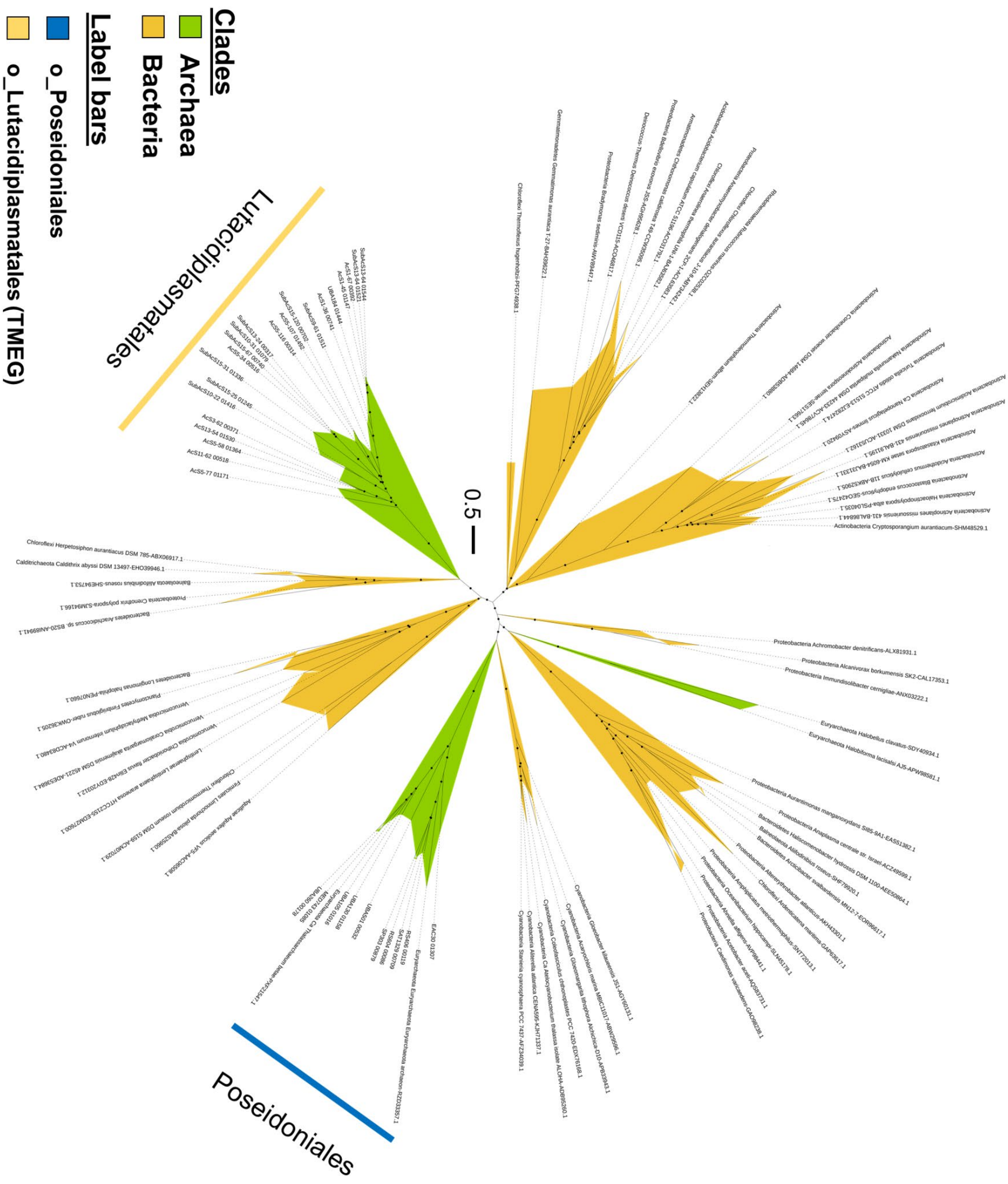

Supplementary Fig. 11. Phylogeny of the heme A synthase (*cta*A) gene. Dots indicate branches with ≥ 70% of 1,000 UFBoot replicates. The tree was estimated using the model LG+F+R7.

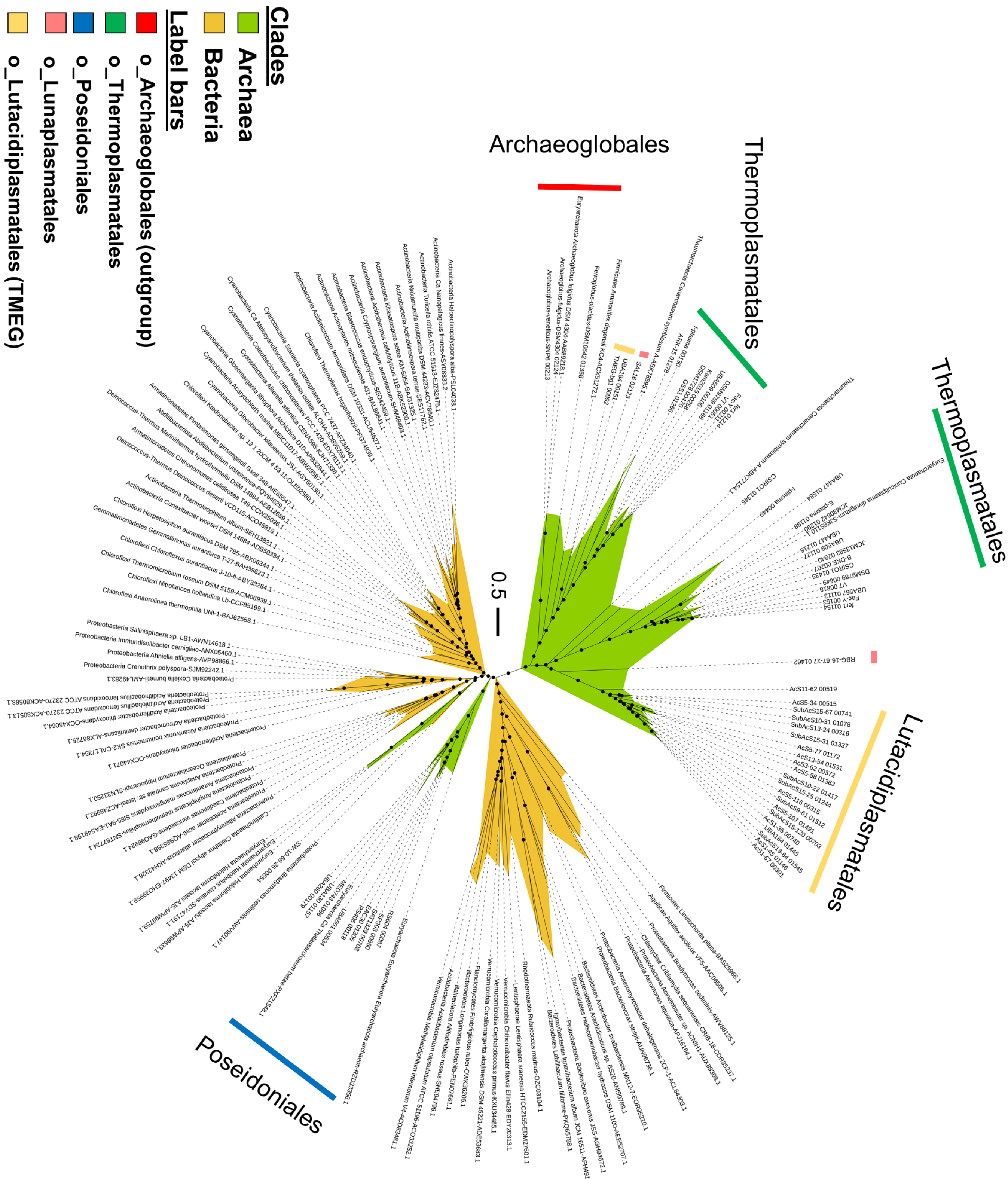

Supplementary Fig. 12. Phylogeny of the protoheme IX farnesyltransferase (*cta*B) gene. Dots indicate branches with ≥ 70% of 1,000 UFBoot replicates. The tree was estimated using the model LG+F+R

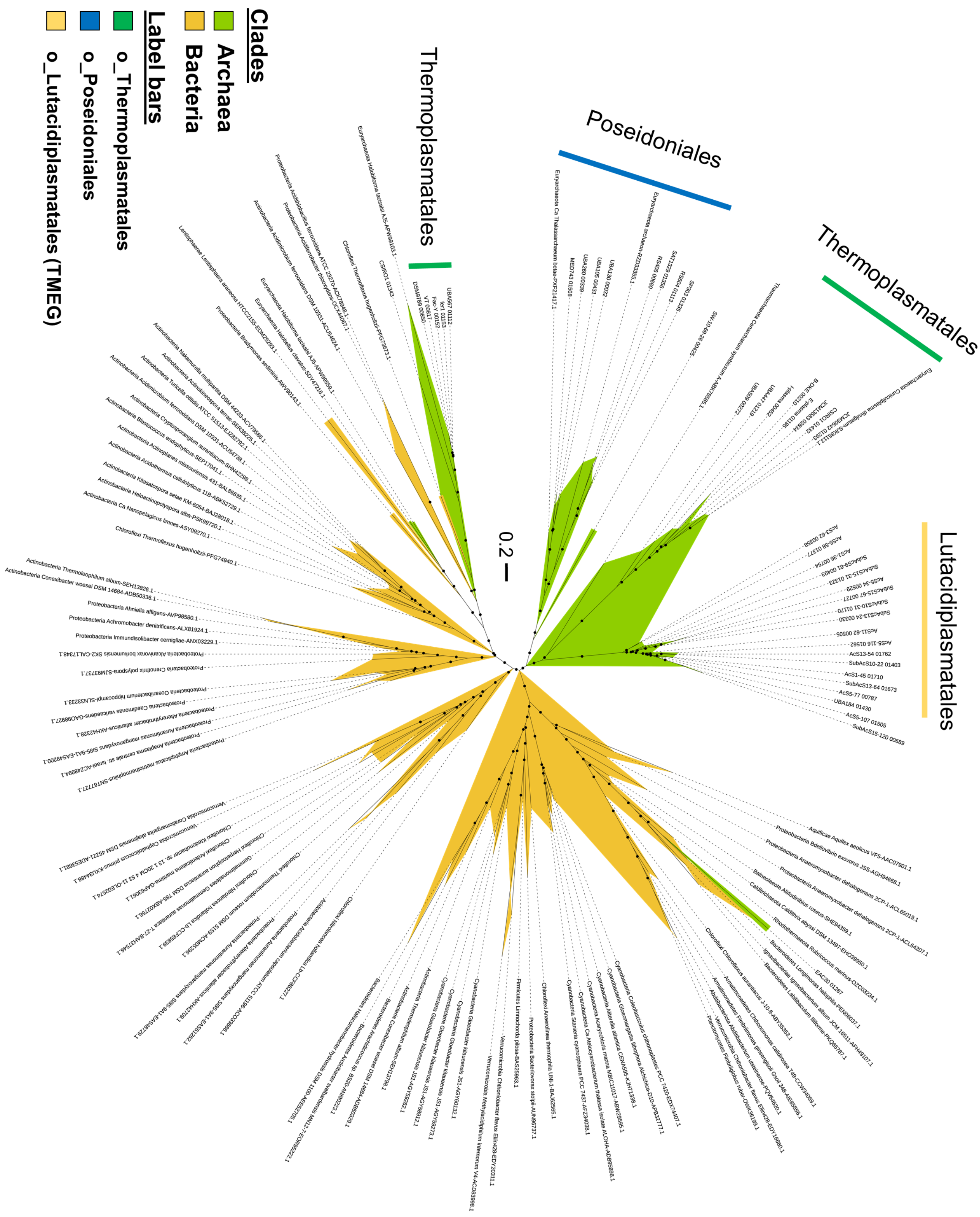

Supplementary Fig. 13. Phylogeny of the heme-copper oxygen reductase subunit B (*cox*B) gene. Dots indicate branches with ≥ 70% of 1,000 UFBoot replicates. The tree was estimated using the model LG+R6.

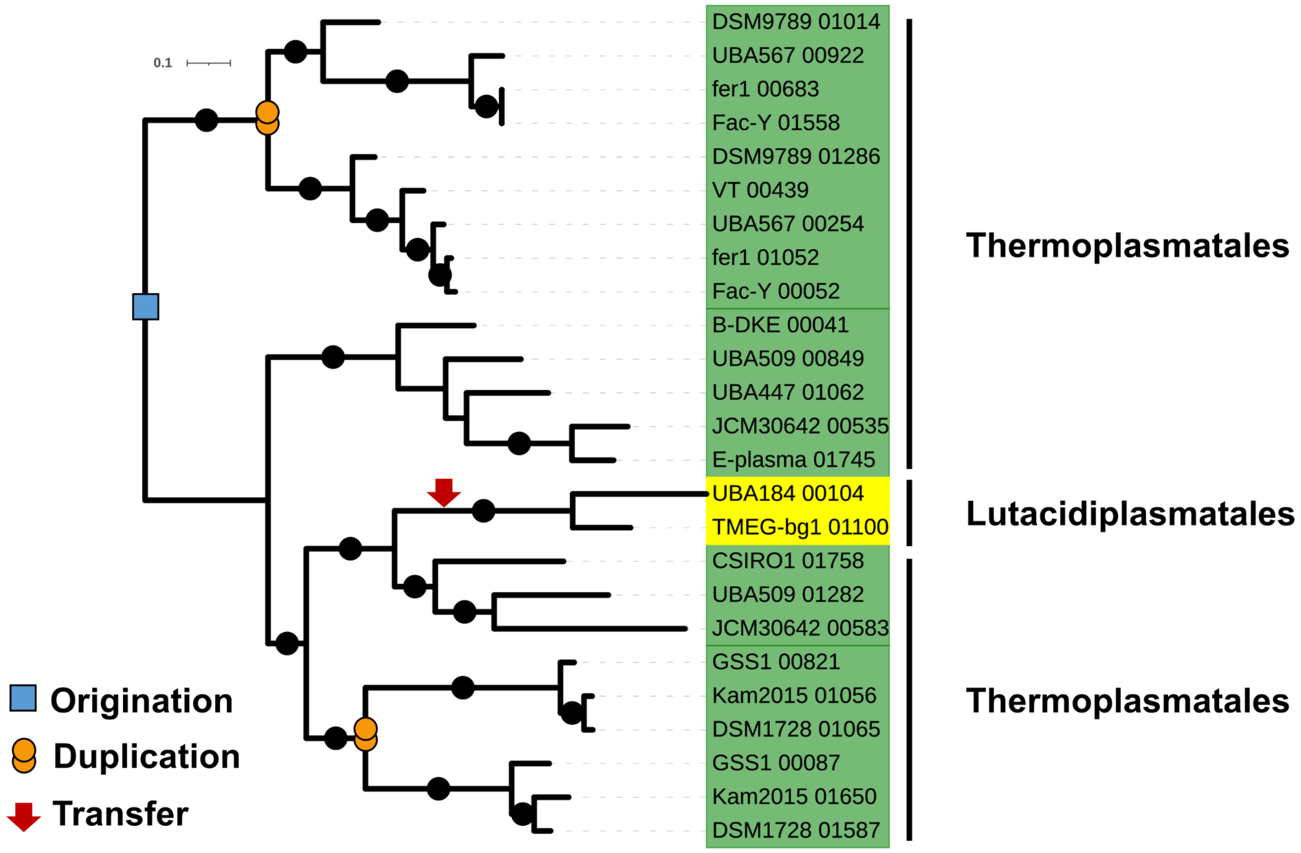

Supplementary Fig. 14. Evolutionary history of the cytochrome bd ubiquinol oxidase (*cyd*A) gene in Thermoplasmatota. The *cyd*A gene is predicted to have been acquired by lateral gene transfer by a Thermoplasmatales ancestor. The gene was subsequently duplicated within the Thermoplasmatales and laterally transferred to the LCA of Lutacidiplasmatales genomes, UBA184 and TMEG-bg1. The *cyd*A gene phylogenetic tree was estimated using the LG+F+G4 model and rooted using minimal ancestor deviation (MAD). Dots indicate branches with ≥ 70% of 1,000 UFBoot replicates.

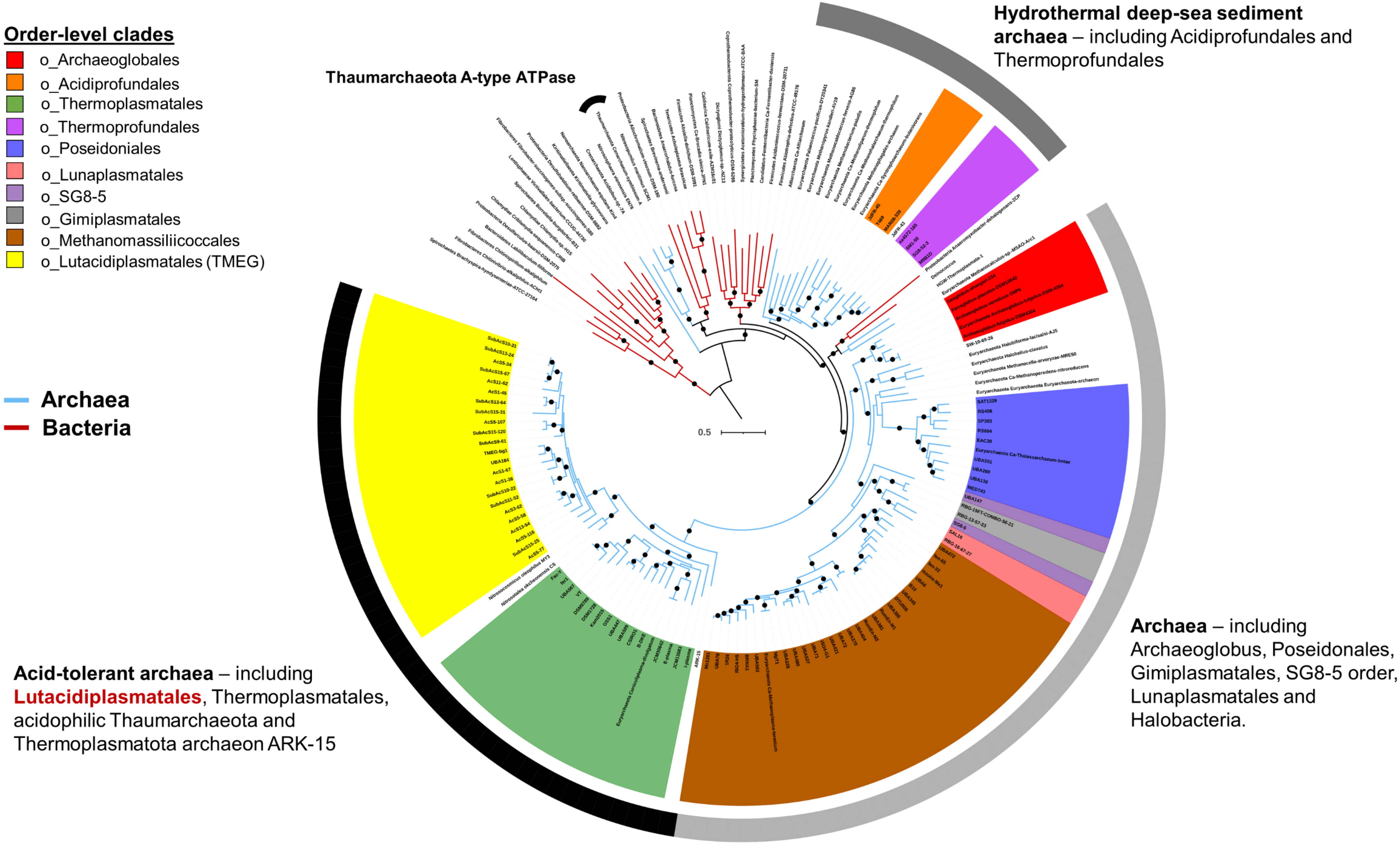
Order-level clades
o_Archaeoglobales
o_Acidiprofundales
o_Thermoplasmatales
o_Thermoprofundales
o_Poseidoniales
o_Lunaplasmatales
o_SG8-5
o_Gimiplasmatales
o_Methanomassiliicoccales
o_Lutacidiplasmatales (TMEG)
Archaea
Bacteria
Thaumarchaeota A-type ATPase
0.5
Hydrothermal deep-sea sediment archaea – including Acidiprofundales and Thermoprofundales
Archaea – including Archaeoglobus, Poseidonales, Gimiplasmatales, SG8-5 order, Lunaplasmatales and Halobacteria.
Acid-tolerant archaea – including Lutacidiplasmatales, Thermoplasmatales, acidophilic Thaumarchaeota and Thermoplasmatota archaeon ARK-15

Supplementary Fig. 15. Phylogeny of the V/A-ATPase. Lutacidiplasmatales cluster with the acid-tolerant archaea. The three largest subunits of V/A-ATPase (*atp*A, B and I) were individually aligned and then concatenated into a single partitioned supermatrix. A supermatrix tree was then estimated using the best fitting model for each partition and rooted using minimal ancestor deviation (MAD). Dots indicate branches with ≥ 70% of 1,000 UFBoot replicates.

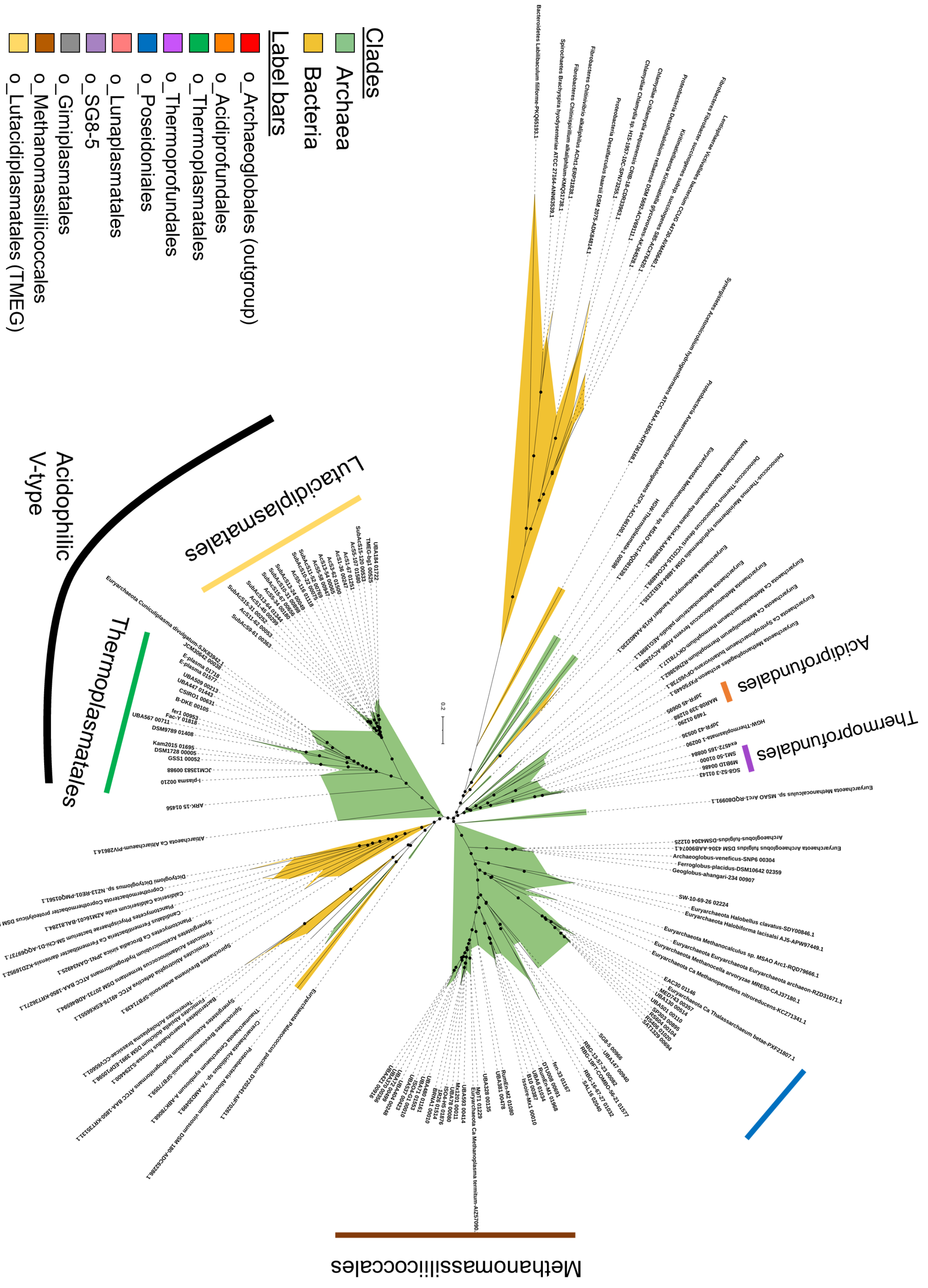

Supplementary Fig. 16. Phylogeny of the V/A-ATPase subunit A (*atp*A) gene. Dots indicate branches with ≥ 70% of 1,000 UFBoot replicates. The tree was estimated using the model LG+R6.

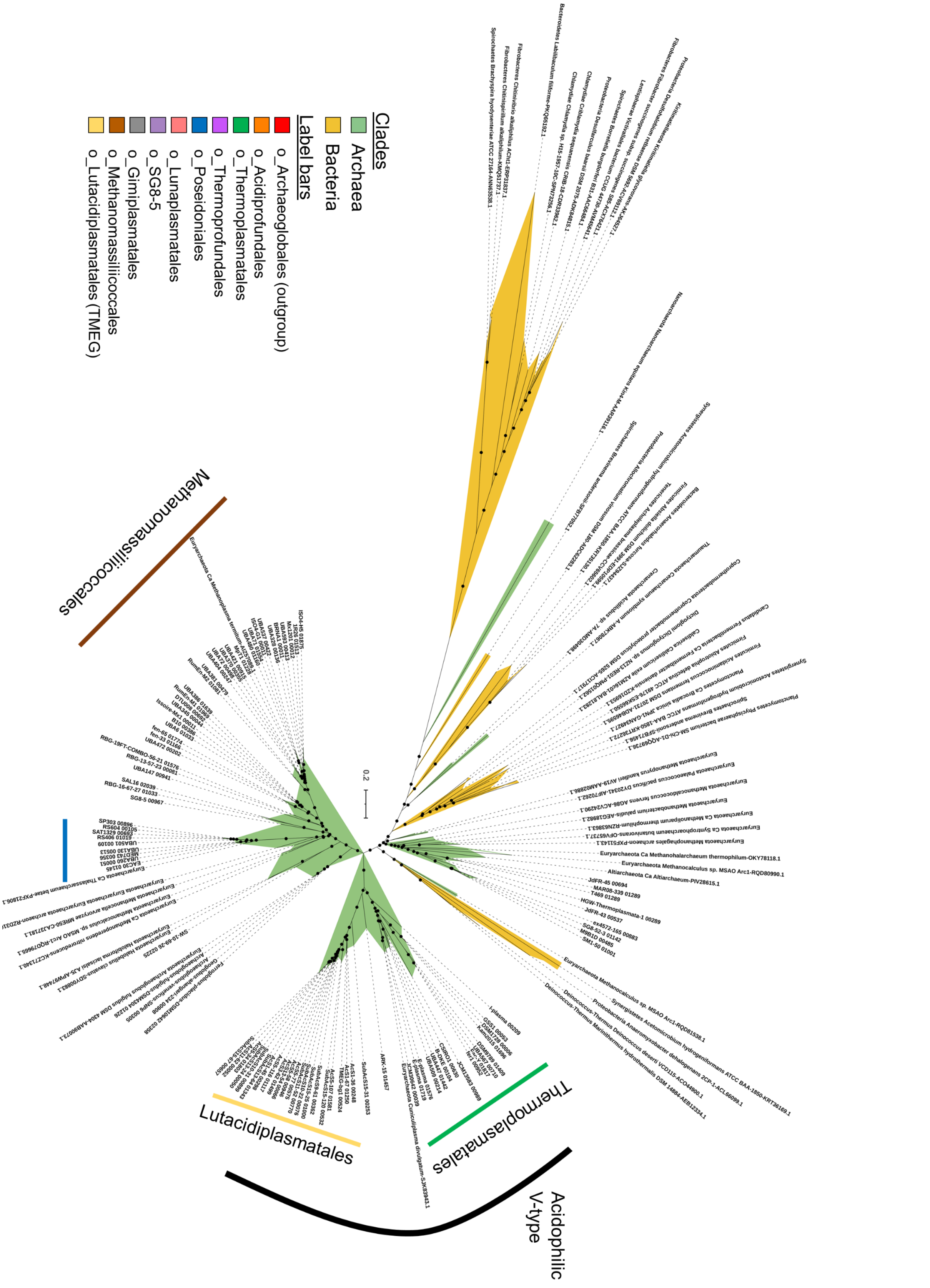

Supplementary Fig. 17. Phylogeny of the V/A-ATPase subunit B (*atp*B) gene. Dots indicate branches with ≥ 70% of 1,000 UFBoot replicates. The tree was estimated using the model LG+R6.

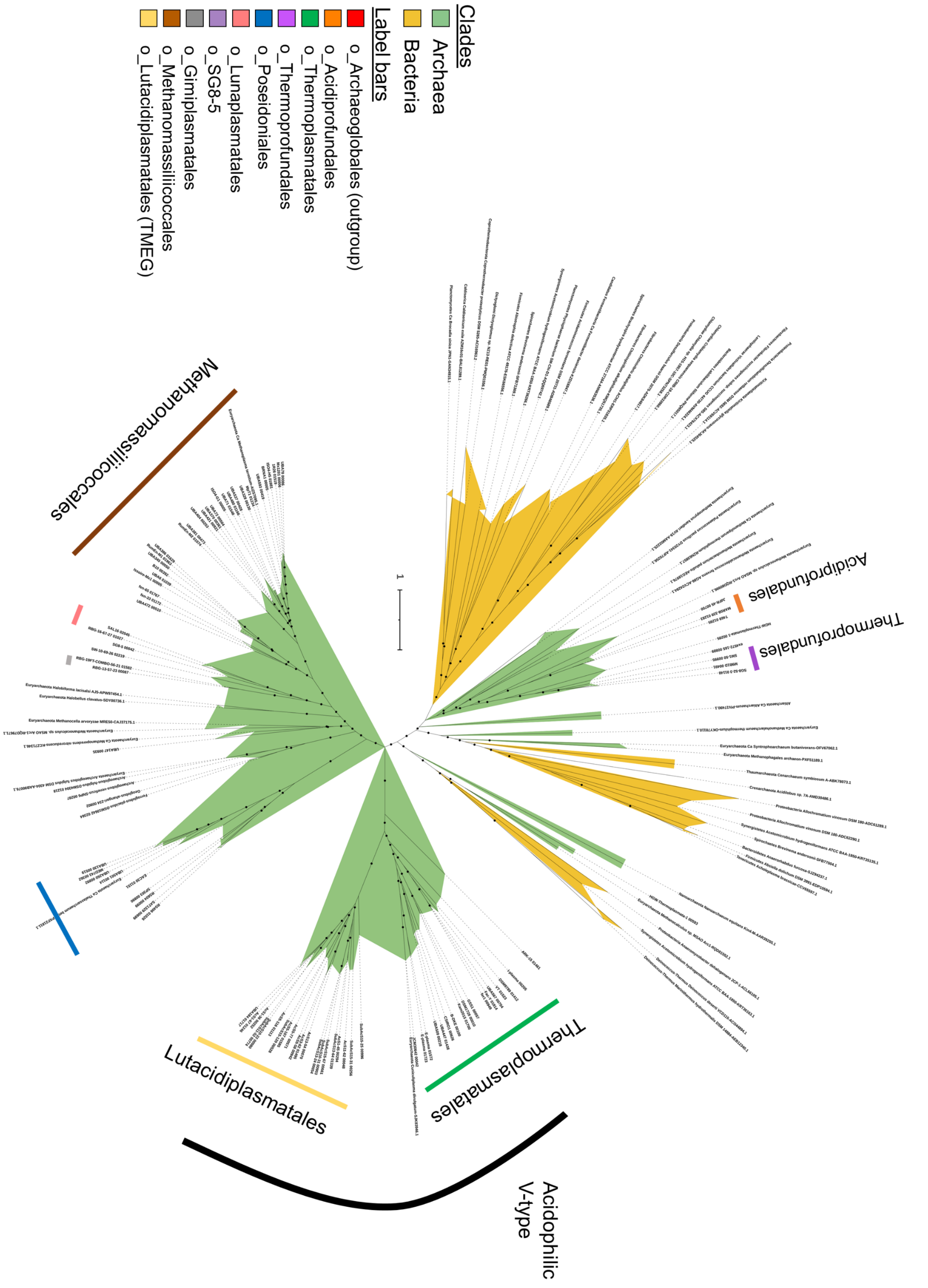

Supplementary Fig. 18. Phylogeny of the V/A-ATPase subunit I (*atp*I) gene. Dots indicate branches with ≥ 70% of 1,000 UFBoot replicates. The tree was estimated using the model LG+F+R7.

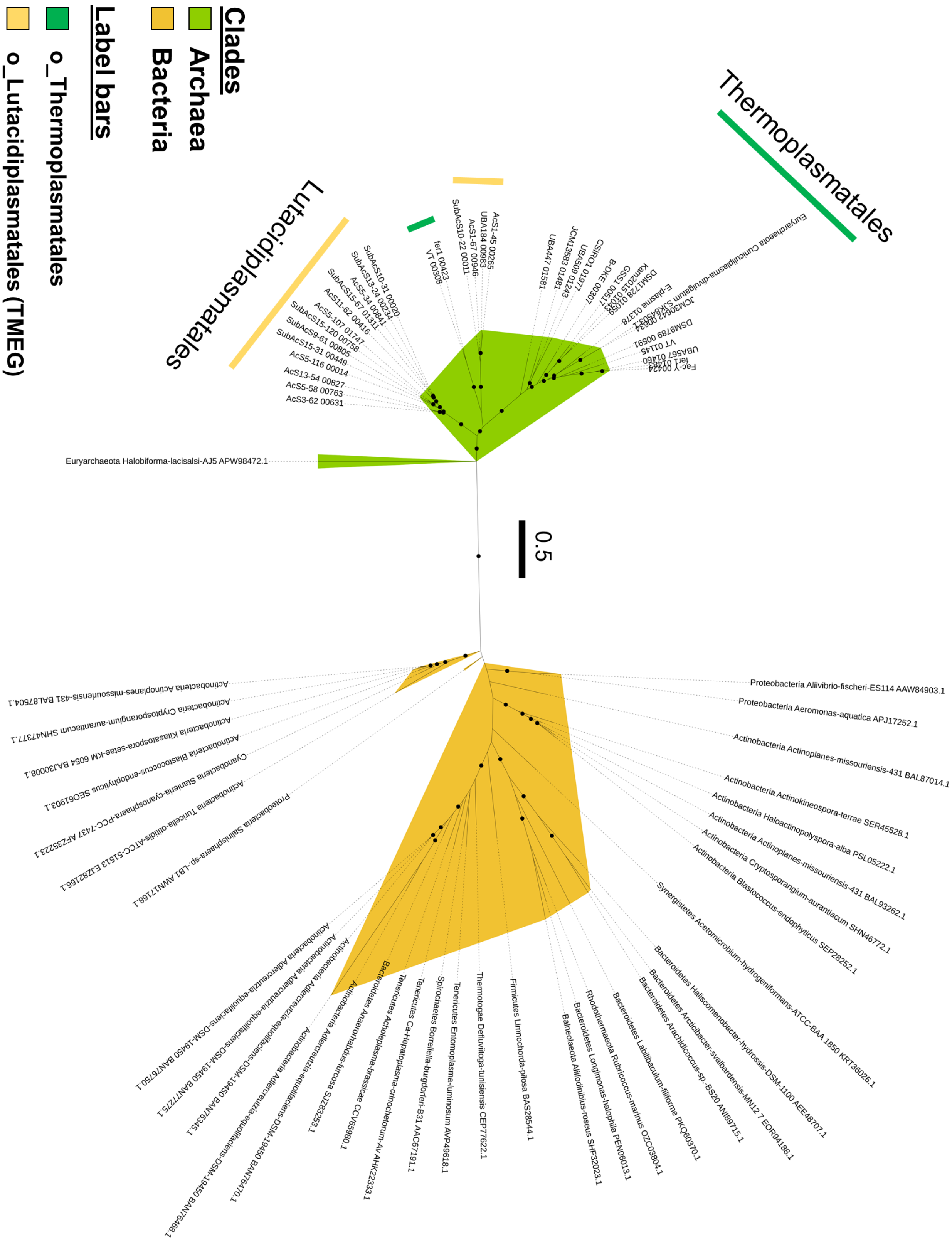

Supplementary Fig. 19. Phylogeny of the arginine deiminase (*arc*A) gene. Dots indicate branches with ≥ 70% of 1,000 UFBoot replicates. The tree was estimated using the mode LG+R5.

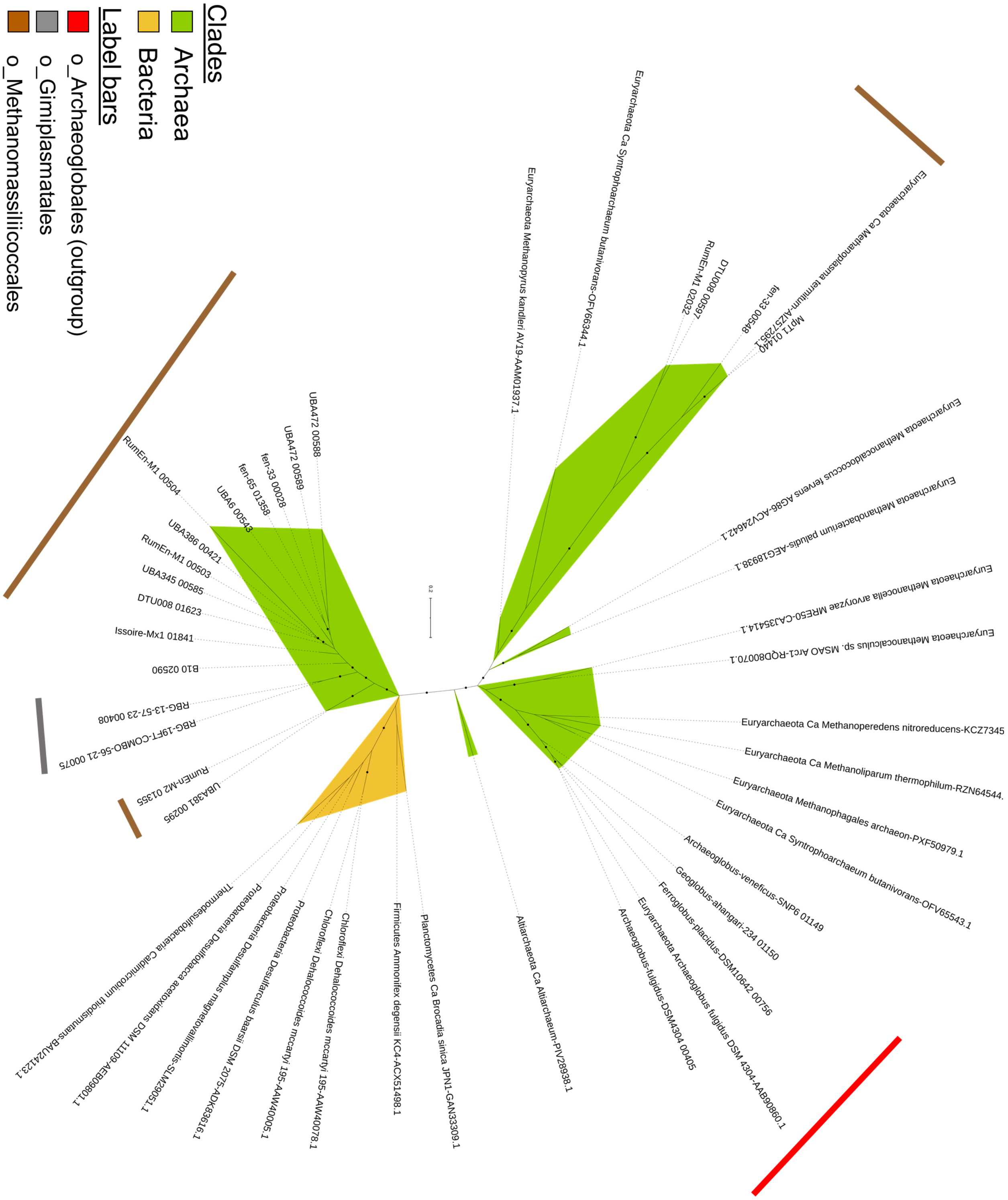

Supplementary Fig. 20. Phylogeny of the CO dehydrogenase/acetyl-CoA synthase complex subunit gamma (*cdh*E). Dots indicate branches with ≥ 70% of 1,000 UFBoot replicates. The tree was estimated using the model LG+I+G4.

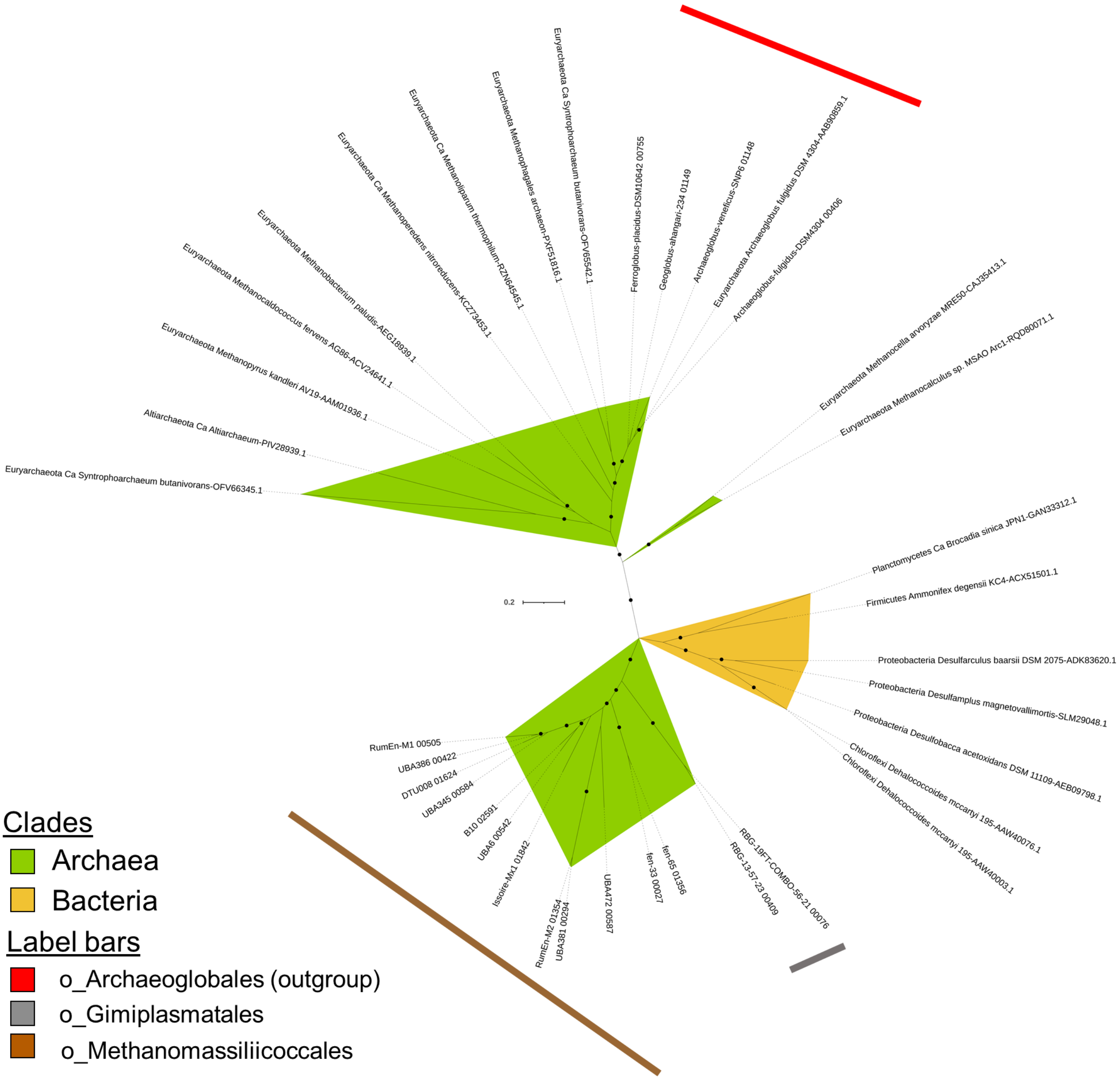

Supplementary Fig. 21. Phylogeny of the CO dehydrogenase/acetyl-CoA synthase complex subunit delta (*cdh*D). Dots indicate branches with ≥ 70% of 1,000 UFBoot replicates. The tree was estimated using the model LG+I+G4.

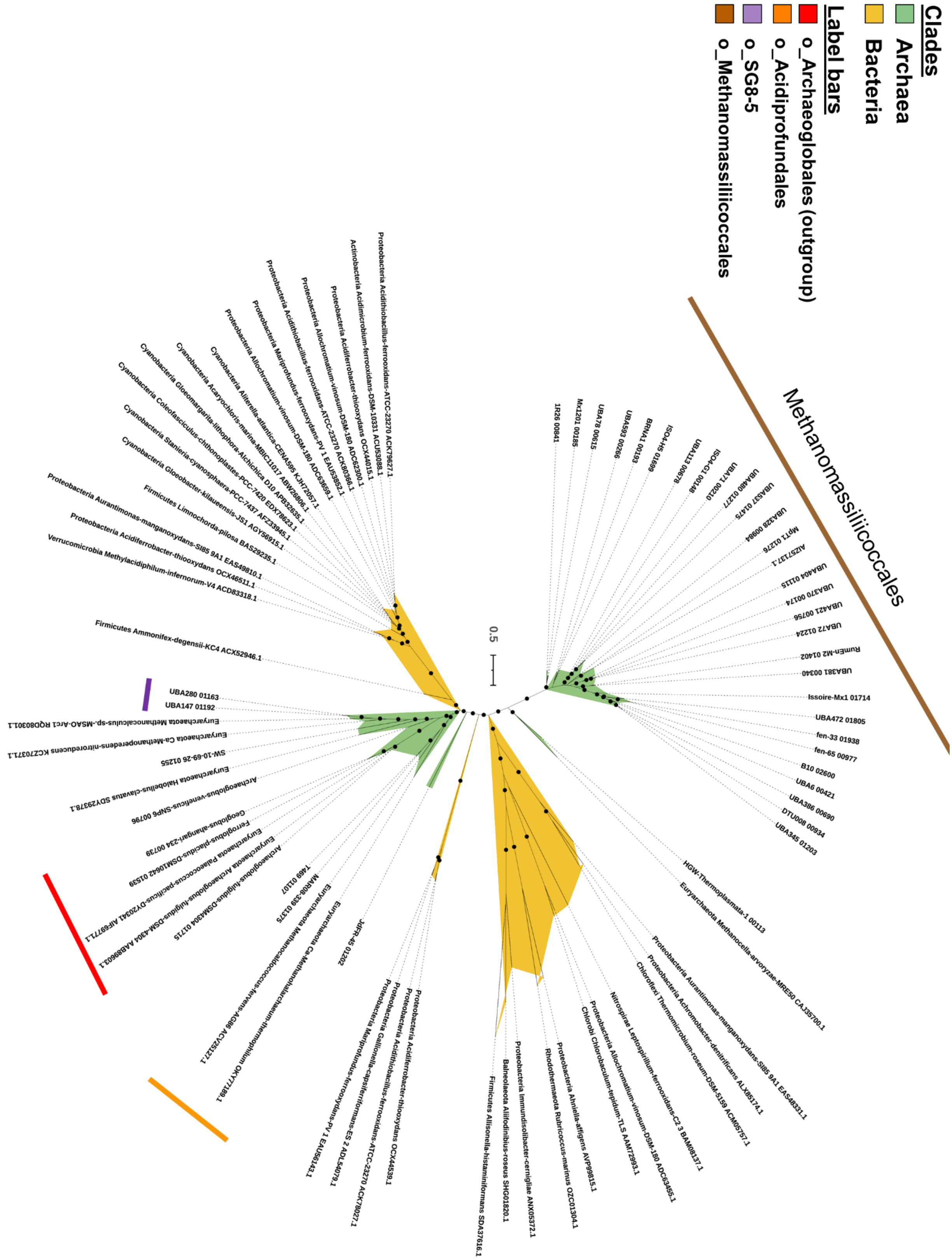

Supplementary Fig. 22. Phylogeny of the ribulose bisphosphate carboxylase, large chain subunit (*rbc*L). Dots indicate branches with ≥ 70% of 1,000 UFBoot replicates. The tree was estimated using the model LG+R5.

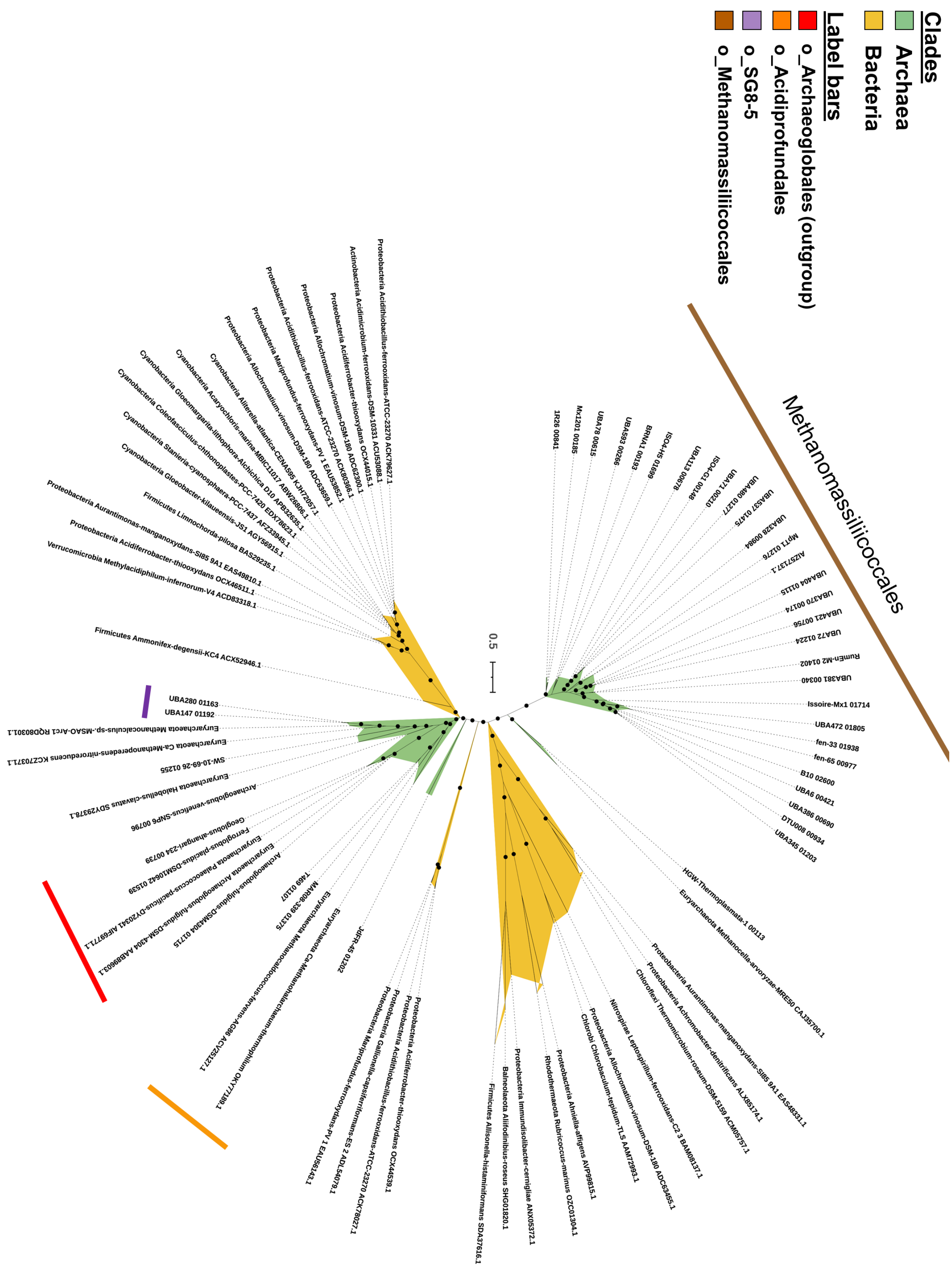

Supplementary Fig. 23. Phylogeny of the ATP-dependent phosphofructokinase (*pfk*). Dots indicate branches with ≥ 70% of 1,000 UFBoot replicates. The tree was estimated using the model LG+R6.

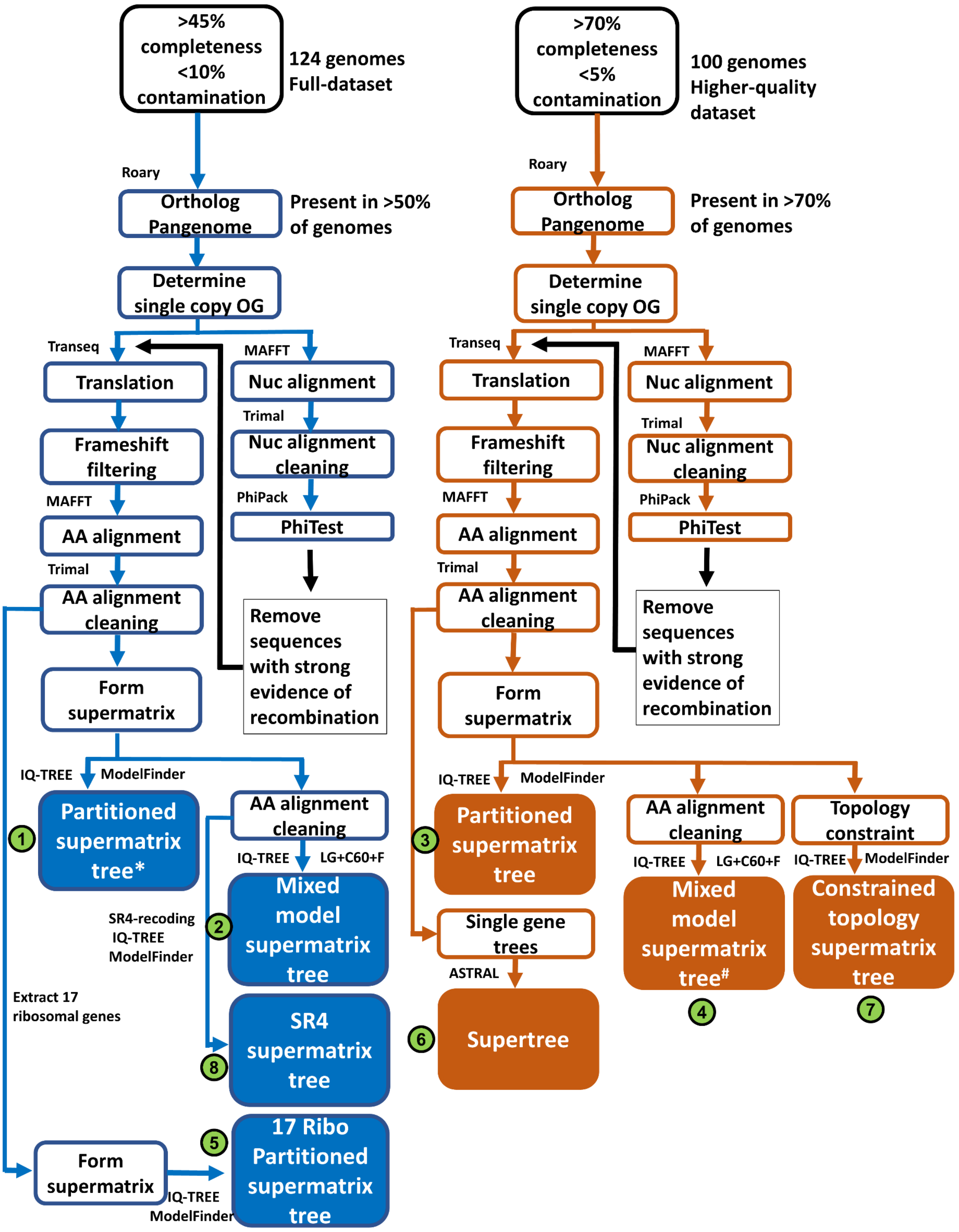

Supplementary Fig. 24. Schematic workflow of construction of eight phylogenomic trees estimating Thermoplasmatota evolutionary relationships. Tools used in the blue and orange branches of the workflow are shown next to their stage of use. The numbered colour-filled boxes indicate the eight trees created in this workflow. The asterisk (*) indicates the tree used in the creation of Fig. 2, whereas the hashtag (#) indicates the tree used as a species tree in the gene tree-species tree reconciliation. The green numbers (1 to 8) correspond to the tree numbers presented in Supplementary Fig. 4. The number of marker genes and supermatrix size for each tree is described in Supplementary Data 26.

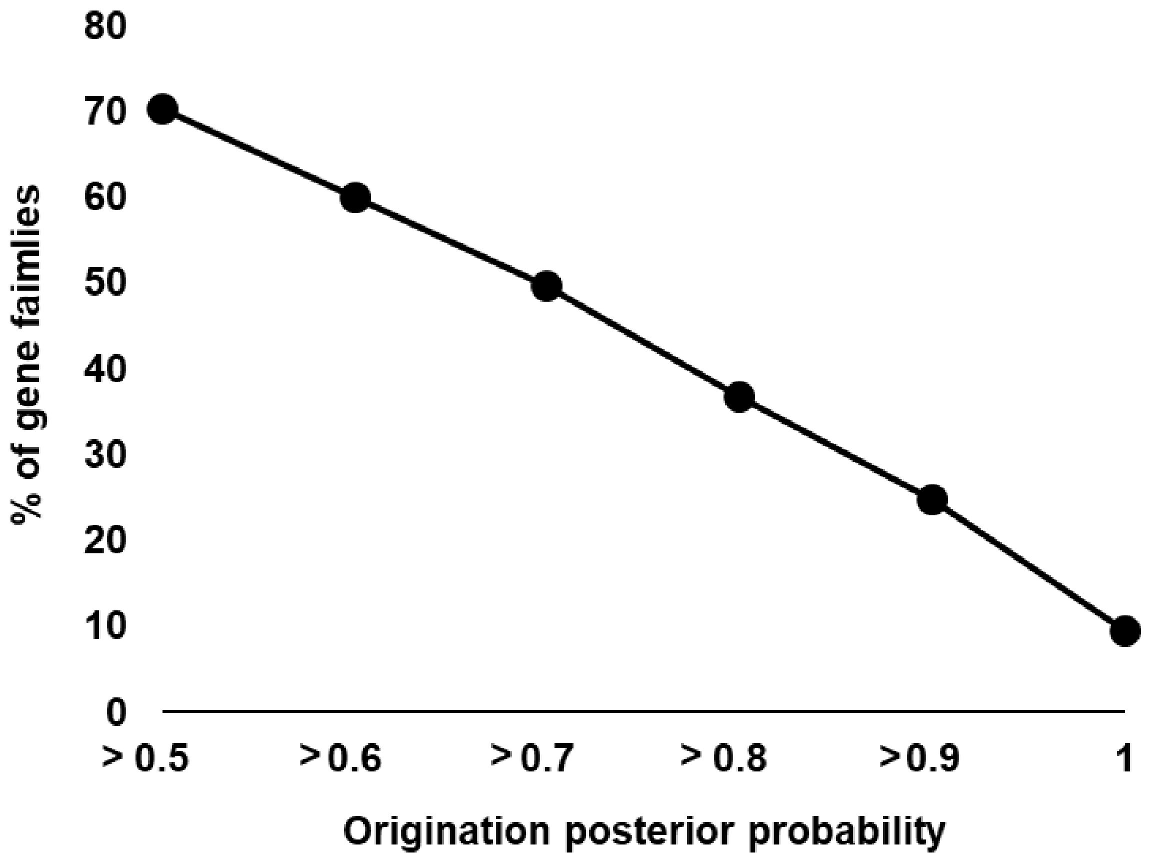

Supplementary Fig. 25. Percentage of the 6,050 gene families predicted to have a single point of origination against increasing stringency of the posterior probability. There is a linear decrease in the percentage of gene families with increasing origination posterior probability.

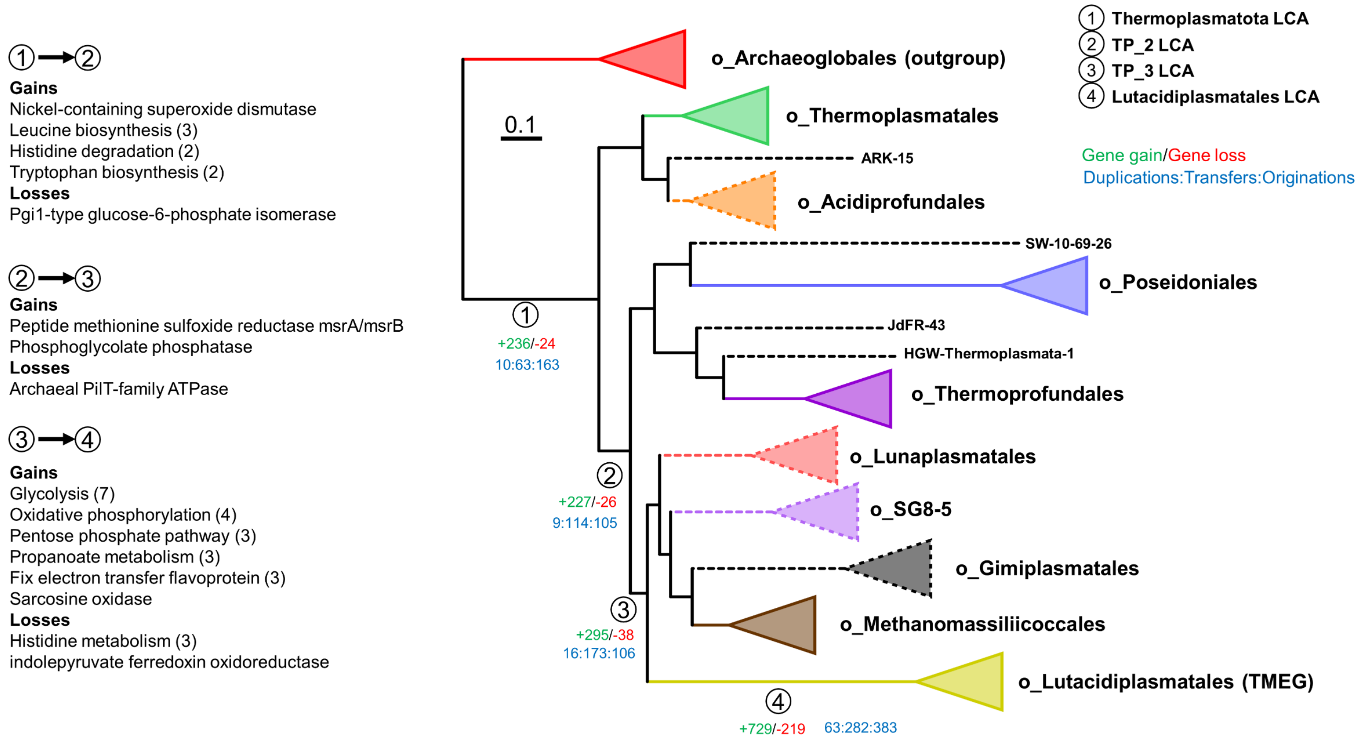

Supplementary Fig. 26. Gene content evolution from the Thermoplasmatota LCA to Lutacidiplasmatales LCA. The gain and loss of gene families between the ancestral gene content reconstructions of the Thermoplasmatota LCA (1), the first divergence (2. TP_2 LCA), the second divergence (3. TP_3 LCA) and the final divergence to the Lutacidiplasmatales LCA (4). The triangles represent collapsed clades, and the dotted triangles indicate that the clade consists of less than four representative genomes. The number of genes gained or lost for the listed metabolisms is conveyed in parenthesis.

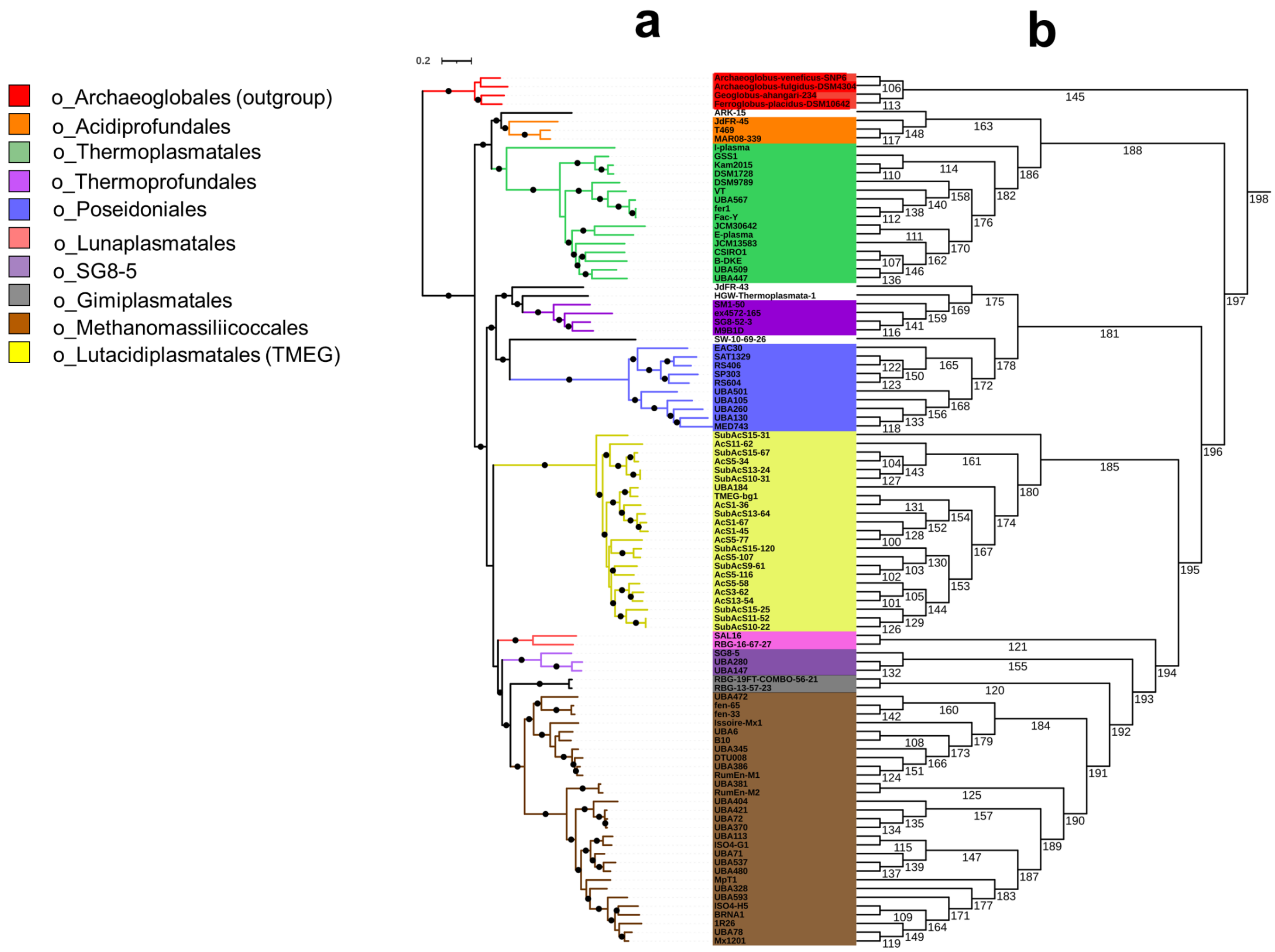

Supplementary Fig. 27. Higher-quality genome dataset tree(a) and branch labelled (b) phylogeny of Thermoplasmatota. For the Higher-quality genome dataset tree (**a**), the ML tree was estimated using the best fitting model for each of the 71 concatenated genes. This species tree was used as input for the gene tree-species tree reconciliation. Dots indicate branches with >70% UFBoot and SH-aLRT support. The cladogram (**b**) possesses the topology of the species tree presented and is annotated with the branch numbers referred to in Supplementary Data 13. These two datasets are combined in Fig. 3.

Supplementary References

1. Sheridan, P. O. *et al*. Gene duplication drives genome expansion in a major lineage of Thaumarchaeota. *Nature communications* **11**, 1-12 (2020).

2. Jones, P. *et al*. InterProScan 5: genome-scale protein function classification. *Bioinformatics* **30**, 1236-1240 (2014).

3. El-Gebali, S. *et al*. The Pfam protein families database in 2019. *Nucleic Acids Res.* **47**, D427-D432 (2019).

4. Gough, J., Karplus, K., Hughey, R. & Chothia, C. Assignment of homology to genome sequences using a library of hidden Markov models that represent all proteins of known structure. *J. Mol. Biol.* **313**, 903-919 (2001).

5. Necci, M., Piovesan, D., Dosztányi, Z. & Tosatto, S. C. MobiDB-lite: fast and highly specific consensus prediction of intrinsic disorder in proteins. *Bioinformatics* **33**, 1402-1404 (2017).

6. Krogh, A., Larsson, B., Von Heijne, G. & Sonnhammer, E. L. Predicting transmembrane protein topology with a hidden Markov model: application to complete genomes. *J. Mol. Biol.* **305**, 567-580 (2001).

7. Katoh, K., Misawa, K., Kuma, K. & Miyata, T. MAFFT: a novel method for rapid multiple sequence alignment based on fast Fourier transform. *Nucleic Acids Res.* **30**, 3059-3066 (2002).

8. Capella-Gutiérrez, S., Silla-Martínez, J. M. & Gabaldón, T. trimAl: a tool for automated alignment trimming in large-scale phylogenetic analyses. *Bioinformatics* **25**, 1972-1973 (2009).

9. Nguyen, L., Schmidt, H. A., Von Haeseler, A. & Minh, B. Q. IQ-TREE: a fast and effective stochastic algorithm for estimating maximum-likelihood phylogenies. *Mol. Biol. Evol.* **32**, 268-274 (2015).

10. Guindon, S. *et al*. New algorithms and methods to estimate maximum-likelihood phylogenies: assessing the performance of PhyML 3.0. *Syst. Biol.* **59**, 307-321 (2010).

11. Kalyaanamoorthy, S., Minh, B. Q., Wong, T. K., von Haeseler, A. & Jermiin, L. S. ModelFinder: fast model selection for accurate phylogenetic estimates. *Nature methods* **14**, 587 (2017).

12. Tria, F. D. K., Landan, G. & Dagan, T. Phylogenetic rooting using minimal ancestor deviation. *Nature ecology & evolution* **1**, 1-7 (2017).

13. Adam, P. S., Borrel, G., Brochier-Armanet, C. & Gribaldo, S. The growing tree of Archaea: new perspectives on their diversity, evolution and ecology. *The ISME journal* **11**, 2407-2425 (2017).

14. Hu, W. *et al*. Metagenomic insights into the metabolism and evolution of a new Thermoplasmata order (Candidatus Gimiplasmatales). *Environ. Microbiol.* (2020).

15. Diamond, S. *et al*. Soils and sediments host Thermoplasmata archaea encoding novel copper membrane monooxygenases (CuMMOs). *The ISME Journal*, 1-15 (2022).

16. Mai, X. & Adams, M. Indolepyruvate ferredoxin oxidoreductase from the hyperthermophilic archaeon Pyrococcus furiosus. A new enzyme involved in peptide fermentation. *J. Biol. Chem.* **269**, 16726-16732 (1994).

17. Narrowe, A. B. *et al*. Complex evolutionary history of translation elongation factor 2 and diphthamide biosynthesis in archaea and parabasalids. *Genome biology and evolution* **10**, 2380-2393 (2018).

18. Lagkouvardos, I. *et al*. IMNGS: a comprehensive open resource of processed 16S rRNA microbial profiles for ecology and diversity studies. *Scientific reports* **6**, 1-9 (2016).

19. Li, G., Rabe, K. S., Nielsen, J. & Engqvist, M. K. Machine learning applied to predicting microorganism growth temperatures and enzyme catalytic optima. *ACS synthetic biology* **8**, 1411-1420 (2019).

20. Kratsch, C. & McHardy, A. C. RidgeRace: ridge regression for continuous ancestral character estimation on phylogenetic trees. *Bioinformatics* **30**, i527-i533 (2014).
