# Supplementary material for: Recovery of Lutacidiplasmatales archaeal order genomes suggests convergent evolution in Thermoplasmatota
Source: Nat Commun. 2022 Jul 15;13:4110. doi: 10.1038/s41467-022-31847-7 (PMC9287336; doi:10.1038/s41467-022-31847-7)
Supplement: Supplementary file 3 — Description of Additional Supplementary Files [file 41467_2022_31847_MOESM3_ESM.pdf]

Description of Additional Supplementary Files

File Name: Supplementary Data 1
Description: Soil characteristics of the eleven sampled soils

File Name: Supplementary Data 2
Description: Genome descriptions

File Name: Supplementary Data 3
Description: Pairwise comparison of average amino acid identity of Lutacidiplasmatales genomes

File Name: Supplementary Data 4
Description: Pairwise comparison of 16S rRNA gene similarity of Lutacidiplasmatales genomes

File Name: Supplementary Data 5
Description: Lutacidiplasmatales genomes as type material for reclassification

File Name: Supplementary Data 6
Description: BLASTn comparsion of AcS3-62 16S rRNA gene against the nonredundant database (June 2020)

File Name: Supplementary Data 7
Description: Presence of Lutacidiplasmataceae and Lutacidiplasma in >150,000 16S rRNA libraries

File Name: Supplementary Data 8
Description: Key pathways in Thermoplasmatota genomes and ancestral reconstructions

File Name: Supplementary Data 9
Description: Comparison of Lutacidiplasmatales oxidative phosphorylation loci to other Thermoplasmatota

File Name: Supplementary Data 10
Description: Carbohydrate active enzymes (CAZymes) of the Thermoplasmatota

File Name: Supplementary Data 11
Description: . Secreted peptidases of the Thermoplasmatota

File Name: Supplementary Data 12
Description: Comparing different species tree estimations using constrained trees

File Name: Supplementary Data 13
Description: Quantified mechanisms of gene content change

File Name: Supplementary Data 14
Description: arCOG annotations of gene family origination hot spots in LCA of Lutacidiplasmatales and Poseidoniales

File Name: Supplementary Data 15

Description: GenBank accessions of phylogenetically diverse reference genomes

File Name: Supplementary Data 16
Description: Comparing different gene tree estimations using constrained trees

File Name: Supplementary Data 17
Description: Reconciliation statistics of cydA gene family

File Name: Supplementary Data 18
Description: Reconciliation statistics of arcA gene family

File Name: Supplementary Data 19
Description: Metagenome sample sequence information

File Name: Supplementary Data 20
Description: Marker gene information for expanded taxa sampling ML trees

File Name: Supplementary Data 21
Description: Probable origination branches of 4,255 gene families

File Name: Supplementary Data 22
Description: Reconciliation statistics of ctaB gene family

File Name: Supplementary Data 23
Description: Progressive K number gains and losses in the evolution from Thermoplasmatota LCA to Lutacidiplasmatales LCA

File Name: Supplementary Data 24
Description: Best hit taxonomy of originating gene families.

File Name: Supplementary Data 25
Description: Duplication and loss of gene families originating in Lutacidiplasmatales in comparison to ancestral gene families

File Name: Supplementary Data 26
Description: Phylogenomic tree data
