# Supplementary material for: Recovery of Lutacidiplasmatales archaeal order genomes suggests convergent evolution in Thermoplasmatota
Source: Nat Commun. 2022 Jul 15;13:4110. doi: 10.1038/s41467-022-31847-7 (PMC9287336; doi:10.1038/s41467-022-31847-7)
Supplement: Supplementary file 5 — Reporting Summary [file 41467_2022_31847_MOESM5_ESM.pdf]

nature portfolio

Corresponding author(s): Cécile Gubry-Rangin

Last updated by author(s): Jun 16, 2022

Reporting Summary

Nature Portfolio wishes to improve the reproducibility of the work that we publish. This form provides structure for consistency and transparency in reporting. For further information on Nature Portfolio policies, see our Editorial Policies and the Editorial Policy Checklist.

Statistics

For all statistical analyses, confirm that the following items are present in the figure legend, table legend, main text, or Methods section.

n/a	Confirmed	
☐	☒	The exact sample size (*n*) for each experimental group/condition, given as a discrete number and unit of measurement
☒	☐	A statement on whether measurements were taken from distinct samples or whether the same sample was measured repeatedly
☒	☐	The statistical test(s) used AND whether they are one- or two-sided<br>*Only common tests should be described solely by name; describe more complex techniques in the Methods section.*
☒	☐	A description of all covariates tested
☒	☐	A description of any assumptions or corrections, such as tests of normality and adjustment for multiple comparisons
☒	☐	A full description of the statistical parameters including central tendency (e.g. means) or other basic estimates (e.g. regression coefficient) AND variation (e.g. standard deviation) or associated estimates of uncertainty (e.g. confidence intervals)
☒	☐	For null hypothesis testing, the test statistic (e.g. *F*, *t*, *r*) with confidence intervals, effect sizes, degrees of freedom and *P* value noted<br>*Give P values as exact values whenever suitable.*
☒	☐	For Bayesian analysis, information on the choice of priors and Markov chain Monte Carlo settings
☒	☐	For hierarchical and complex designs, identification of the appropriate level for tests and full reporting of outcomes
☒	☐	Estimates of effect sizes (e.g. Cohen's *d*, Pearson's *r*), indicating how they were calculated

Our web collection on statistics for biologists contains articles on many of the points above.

Software and code

Policy information about availability of computer code

Data collection	No software was used for data collection.
Data analysis	Custom scripts have been deposited at https://github.com/Tancata/phylo/tree/master/ALE and https://github.com/SheridanPO/ALE_analysis. Open source software used in analysis is referenced in Materials and Methods: MEGAHIT v1.1.3, bwa-mem v0.7.17, MaxBin2, metaBAT2, Prokka v1.14, CheckM v1.1.2, QUAST v5.0.2, Tome v2.0, Barrnap v0.9, GTDB-Tk v1.7.0, Roary v3.12.0, MAFFT v7.407, Trimal v1.4.1, PHItest v1.1, IQ-TREE v2.0.3, ALE v1.0, iTOL, CompareM v0.1.1, GhostKOALA v2.2, HMMER v3.2.1, CoverM v0.6.1, Diamond v0.9.28, BLASTn v2.9.0., tRNAscan-SE v2.0.5

For manuscripts utilizing custom algorithms or software that are central to the research but not yet described in published literature, software must be made available to editors and reviewers. We strongly encourage code deposition in a community repository (e.g. GitHub). See the Nature Portfolio guidelines for submitting code & software for further information.

Data

Policy information about availability of data

All manuscripts must include a data availability statement. This statement should provide the following information, where applicable:

- Accession codes, unique identifiers, or web links for publicly available datasets
- A description of any restrictions on data availability
- For clinical datasets or third party data, please ensure that the statement adheres to our policy

Accession numbers for the 36 newly sequenced genomes presented in this study can be found in Supplementary Data 2 and under the NCBI BioProject PRJNA795910 (https://www.ncbi.nlm.nih.gov/bioproject/?term=PRJNA795910). Public data is available from NCBI (www.ncbi.nlm.nih.gov), IMG (https://img.jgi.doe.gov/), KEGG (https://www.genome.jp/kegg/), dbCAN (http://bcb.unl.edu/dbCAN2/download/), arCOG (https://ftp.ncbi.nih.gov/pub/wolf/COGs/arCOG/), PFAM (https://pfam.xfam.org/), TIGRFAM (http://tigrfams.jcvi.org/cgi-bin/index.cgi) and GTDB R202 (https://data.gtdb.ecogenomic.org/releases/).

Human research participants

Policy information about studies involving human research participants and Sex and Gender in Research.

Reporting on sex and gender	N/A
Population characteristics	N/A
Recruitment	N/A
Ethics oversight	N/A

Note that full information on the approval of the study protocol must also be provided in the manuscript.

Field-specific reporting

Please select the one below that is the best fit for your research. If you are not sure, read the appropriate sections before making your selection.

☐ Life sciences ☐ Behavioural & social sciences ☒ Ecological, evolutionary & environmental sciences

For a reference copy of the document with all sections, see nature.com/documents/nr-reporting-summary-flat.pdf

Ecological, evolutionary & environmental sciences study design

All studies must disclose on these points even when the disclosure is negative.

Study description	Presentation and genomic analysis of novel metagenome-assembled genomes sequences from the Terrestrial Miscellaneous Euryarchaeota Group archaea and evolutionary analysis of the Thermoplasmatota phylum by gene tree - species tree reconciliation techniques.
Research sample	Analysis involved 120 Thermoplasmatota genome sequences (including 36 novel genomes from this study) and four Archaeoglobales genomes. The 36 novel genome sequences were sequenced from acidic soils collected around Scotland, UK. The publicly available genome sequences were sequenced from a wide variety of environmental sources, including marine, soil and hot spring environments. The rationale of this sampling was intended to represent the Thermoplasmatota phylum with related outgroups, so that genome evolution in the major lineages of Thermoplasmatota could be investigated, with a particular emphasis on the novel order Lutacidiplasmatales.
Sampling strategy	Gene duplication, transfer, loss and origination data was sampled from 98 branches. This sample size is the total number of internal branches on the Thermoplasmatota species tree used for gene tree-species tree reconcilition.
Data collection	Public Thermoplasmatota genome sequences were downloaded from IMG (https://img.jgi.doe.gov/) and NCBI (www.ncbi.nlm.nih.gov) by Dr Paul O. Sheridan onto a local computer system.
Timing and spatial scale	Collection of data from public repositories was conducted in August 2021. No further public genomes were utilised after this date, as due to the nature of the data analysis in this study (the formation of gene families, construction of gene trees and reconciliation against species trees) it was not possible to add additional genomes without restarting the entire analysis.
Data exclusions	Thermoplasmatota genomes with a completeness lower than 45 % or contamination greater than 10 % were excluded from the study. These thresholds were chosen specifically for this dataset.
Reproducibility	Not fully applicable as this was a data analysis study, rather than experimental observation, but the code and databases used to analyze data in the study can be accessed as described in the code and data availability sections.

Randomization	The genomes were analyzed as a single group without partitions. Genes encoded by genomes were related to each other by amino acid sequence information, rather than by any a priori clustering and can thus be considered to be randomized.
Blinding	The genomes were analyzed as a single group with no a priori clustering. While the researchers were not blind to the taxonomy of the genomes, this information was not provided to the phylogenetic that was used to infer the phylogenies of genes and species.

Did the study involve field work? ☐ Yes ☒ No

Reporting for specific materials, systems and methods

We require information from authors about some types of materials, experimental systems and methods used in many studies. Here, indicate whether each material, system or method listed is relevant to your study. If you are not sure if a list item applies to your research, read the appropriate section before selecting a response.

Materials & experimental systems

n/a	Involved in the study
☒	☐ Antibodies
☒	☐ Eukaryotic cell lines
☒	☐ Palaeontology and archaeology
☒	☐ Animals and other organisms
☒	☐ Clinical data
☒	☐ Dual use research of concern

Methods

n/a	Involved in the study
☒	☐ ChIP-seq
☒	☐ Flow cytometry
☒	☐ MRI-based neuroimaging
